# Comparative Analysis of Localization and Composition of Adult Neurogenic Niches in the Chondrichthyans *Raja asterias* and *Torpedo ocellata*

**DOI:** 10.3390/ijms26083563

**Published:** 2025-04-10

**Authors:** Sara Bagnoli, Davide Lorenzo Drago, Emanuele Astoricchio, Elena Chiavacci, Baldassarre Fronte, Alessandro Cellerino, Eva Terzibasi Tozzini

**Affiliations:** 1 Biology Laboratory (BIO@SNS), Scuola Normale Superiore, 56126 Pisa, Italy; sara.bagnoli@sns.it (S.B.); davide.drago@sns.it (D.L.D.); elena.chiavacci@sns.it (E.C.); alessandro.cellerino@sns.it (A.C.); 2 Biology and Evolution of Marine Organisms Department (BEOM), Stazione Zoologica Anton Dohrn, 80121 Napoli, Italy; emanuele.astoricchio@szn.it; 3 Department of Veterinary Sciences, University of Pisa, 56124 Pisa, Italy; baldassarre.fronte@unipi.it

**Keywords:** adult neurogenesis, *Raja asterias*, *Torpedo ocellata*, neurogenic niches, PCNA, S100B

## Abstract

Adult neurogenesis in cartilaginous fish remains a relatively unexplored area, particularly in terms of comparative analysis. This process, defined as the ability of specialized stem cells to generate new functional neurons, has gained prominence due to its relevance in neurodegenerative disease research and regenerative medicine. However, there is an ongoing discussion about when and where it first appeared. Evidence of adult neurogenesis in both teleosts and mammals highlights significant differences, such as the number of newly formed cells and the brain regions involved. Investigating additional cartilaginous fish species, which occupy a basal position in vertebrate phylogeny, could provide valuable insights into the ancient origins of this trait and potentially new general knowledge about the adult neurogenesis process. In this study, we combined immunohistochemistry and in situ hybridization to examine neurogenic activity in three brain regions—the telencephalon, mesencephalon, and cerebellum—of two batoid species: *Raja asterias* and *Torpedo ocellata*. Immunohistochemical methods were used to identify neurogenic cells by employing markers for cell proliferation (PCNA), mitosis (pH3), glial cells (S100B), and stem cells (Msi1). Additionally, in situ hybridization was performed to detect neural stem cell mRNA for *Notch1*, *Notch3*, and *Sox2* in the telencephalon and mesencephalon of *Raja asterias*.

## 1. Introduction

Adult neurogenesis refers to the generation and integration of new functional neurons into the neural circuitry of the adult brain, a process that has garnered significant interest in recent decades [1]. While previously believed to occur exclusively during embryonic development, research has demonstrated that neurogenesis persists at low levels throughout postnatal and adult life in mammals [2,3,4]. In adult mammals, this process is primarily restricted to three regions: the subgranular zone (SGZ) of the hippocampal dentate gyrus [5,6], the subventricular zone (SVZ) of the subpallium [7], and, more recently, the hypothalamic region [8,9,10].

Neurons generated in the SGZ remain within the dentate gyrus and contribute to hippocampal function [11], whereas those produced in the SVZ migrate via the rostral migratory stream to the olfactory bulb, supporting olfactory neuron homeostasis. These neurogenic niches are maintained by glial-like stem cells of embryonic origin, known as radial glial cells (RGs), characterized by their distinctive morphology—a rounded cell body with minimal dendrites and a long radial axon extending through the telencephalic mass [12]. Recent findings also indicate that adult neurogenesis occurs in the hypothalamus, where tanycytes give rise to new neurons [13].

Both embryonic and adult neurogenesis share a similar molecular framework, making them comparable processes. In both cases, RGs generate immature precursors that differentiate into functional neurons [14,15,16,17]. This evolutionary conservation underscores the fundamental significance of neurogenesis in maintaining neural plasticity and function throughout life.

Despite its spatial limitations in mammals, adult neurogenesis appears to be necessary for optimal adult brain function, facilitating the integration of newly generated neurons into existing circuits [18]. Adult neurogenesis is closely related to physiological brain plasticity [19], and reduced levels of neurogenesis have been associated with pathological conditions such as Alzheimer’s disease [20,21]. The activation of radial glial cells and the subsequent production of new neurons are essential for maintaining olfactory bulb homeostasis; studies involving the genetic ablation of these cells in mice have demonstrated a progressive reduction of granule cells in the olfactory bulb, accompanied by impaired contextual and spatial memory, learning, and pattern recognition [22,23].

In the murine hippocampus, the rate of adult neurogenesis varies across the lifespan, with a notable decline observed during aging [21,24]. However, the extent to which hippocampal adult neurogenesis declines with age in humans remains a topic of debate, with some studies suggesting a decline in neurogenesis with age [25], while others indicate that adult neurogenesis or neural progenitor cell (NPC) numbers may not decrease despite a reduction in the progenitor pool [26,27]. Despite the ongoing discussion regarding the decline of neurogenesis in humans, correlations between cognitive status and the number of newborn neurons in both healthy and pathological states suggest that appropriate levels of adult neurogenesis are crucial for maintaining brain functionality [20,28]. Compiled data suggest that new neurons could be derived not only from stem cells but also from a population of neuroblasts displaying a protracted maturation.

Examining adult neurogenesis outside the group of mammals could provide valuable insights into the mechanisms that regulate this process, as the situation varies significantly among other vertebrate groups. Studies on songbirds have demonstrated that adult neurogenesis occurs seasonally in the ventricular zone when they learn new songs, particularly in the hyperstriatum accessorium and hyperstriatum ventralis [29,30]. Various studies have been conducted on different species of bony fish, such as *Danio rerio* [31], the killifish *Nothobranchius furzeri* [32], the knifefish *Apteronotus leptorhynchus*, and the rainbow trout *Oncorhynchus mykiss* [33,34], all demonstrating the presence of several active neurogenic niches localized throughout their brains.

Adult neurogenesis appears to be absent in cyclostomes [35,36], while recent studies in chondrichthyans have revealed extensive neurogenic activity in adult brains [37,38], suggesting that adult neurogenesis could be considered a basal trait of gnathostomes. This observation is particularly interesting because, from a neuroanatomical perspective, chondrichthyans are more similar to mammals than to teleosts. This reasoning stems from the two distinct ways in which telencephalon morphogenesis occurs in these groups: evagination, observed in both chondrichthyans and sarcopterygian tetrapods, versus eversion, commonly seen in actinopterygians, including teleosts [39].

Analyses of the cellular processes of adult neurogenesis and in-depth comparisons of this process between mammals and fish have been extensively reported in the literature (Cacialli and Lucini 2019; Fernández-Hernández and Rhiner 2015; Diotel et al. 2020) [37,40,41,42] and we direct the readers to the cited articles for deeper discussions about this topic.

During evagination, the dorsal pallium folds inward to form a median septum and symmetric lateral ventricles. In contrast, during eversion, the pallium folds outward, causing the area corresponding to the ventricle to extend into the dorso-lateral region [39]. This leads to distinctly different anatomical localizations within the adult telencephalon that can be attributed to the same embryonic origin.

**Figure 1:**
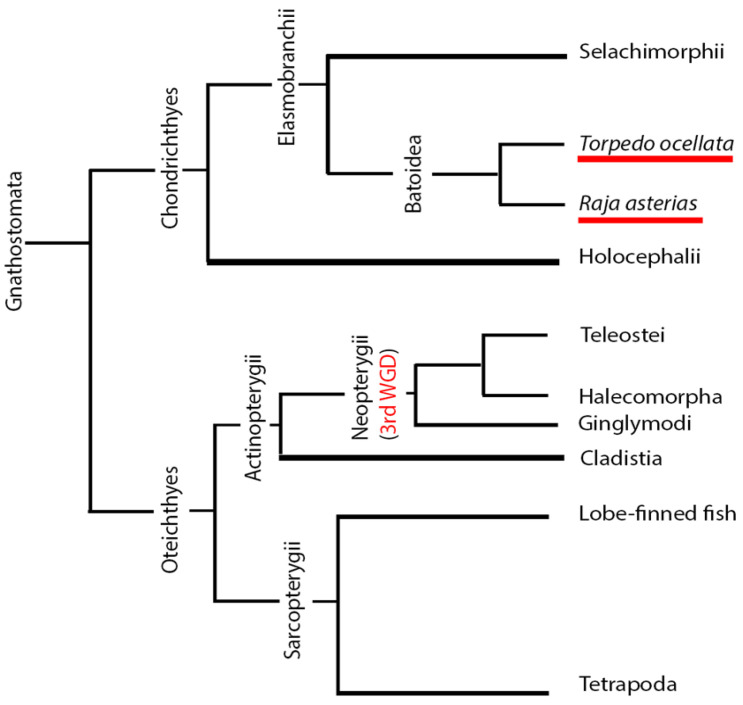
Simplified phylogenetic tree of the *Gnathostomata* group, highlighting the positions of *Raja asterias* and *Torpedo ocellata* (red-underlined). The tree also indicates the positions of the *Teleostei* and *Tetrapoda* clades, as well as the occurrence of the third whole genome duplication event.

The sharing of anatomical features between cartilaginous fishes and mammals can be explained from a phylogenetic perspective (Figure 1). Chondrichthyans are basal to both actinopterygian and tetrapod clades, which can be considered sister groups. Osteichthyes and Chondrichthyes form their own clades that evolved independently from a common Gnathostomata ancestor. Within the Osteichthyes group, the clade of *Sarcopterygii* evolved, from which tetrapods originated. The sister clade to the *Sarcopterygii* is the *Actinopterygii*, from which the *Teleostei* group emerged. Within the *Sarcopterygii* group, tetrapods and teleosts evolved independently, while chondrichthyans are basal to both and have retained many ancestral features. In contrast, actinopterygians, specifically teleosts, have developed numerous unique traits that distinguish them from sarcopterygians. For this reason, some neuroanatomical features of chondrichthyans may be more similar to those of mammals than to those of Osteichthyes. This is one of two main arguments supporting the choice of chondrichthyans as a better alternative model to teleosts for comparative neuroanatomical studies with mammals.

The other important observation supporting this choice is that after the divergence of their clade from that of tetrapods and chondrichthyans, *Actinopterygii* underwent an additional event of whole genome duplication (WGD), further increasing the differences between them and mammals [43] (Figure 1). These arguments validate the use of chondrichthyans as a reference group for studying the evolution of conserved traits such as adult neurogenesis.

The widespread occurrence of adult neurogenesis in the brains of Osteichthyes, contrasted with its absence in tetrapods, raises the question of whether this trait is ancestral and has been lost in tetrapods or if it evolved independently within the Osteichthyes lineage. Recent studies on chondrichthyans may provide insights into this question, and further investigation into the distribution of adult neurogenesis in other chondrichthyan groups is essential to determine whether it can be considered an ancestral feature. To date, insufficient research has been conducted on cartilaginous fishes to definitively ascertain whether adult neurogenesis is a basal trait of gnathostomes.

Currently, the only species in which adult neurogenesis has been characterized is the small-spotted catshark, *Scyliorhinus canicula*, which belongs to the *Galeomorphii* group, a part of the *Selachimorpha* subclass within the class *Elasmobranchii* [37,38]. This class also includes batoideans, which remain poorly characterized in terms of adult neurogenesis.

Considering the lack of knowledge in the context of batoidean adult neurogenesis, in this study, we decided to expand our understanding of this process in *Raja asterias* (R. asterias) and *Torpedo ocellata* (*T. ocellata*). *R. asterias*, commonly known as the Mediterranean starry ray, is a small saltwater fish that reaches a maximum length of 75 cm and is found in the Mediterranean Sea. In contrast, *T. ocellata* is distributed throughout the Mediterranean Sea and along the western coast of Africa, reaching a maximum length of 60 cm.

In general, batoids as rays, skates, and torpedoes are a diverse group of elasmobranchs that exhibit a dorsoventrally compressed body plan. Their nervous system organization presents a unique opportunity to study the evolution of conserved traits, such as adult neurogenesis [44]. Studies on batoid brain morphology and function have revealed adaptations related to their ecological niches, such as larger olfactory bulbs in species inhabiting deep sea or murky waters [45]. Further research on the nervous system of batoids, including *R. asterias* and *T. ocellata*, can provide valuable insights into the evolution and diversity of neural circuits in vertebrates.

The aim of this study on *R. asterias* and *T. ocellata* was to provide a qualitative description and characterization of adult neurogenic niches along the rostro-caudal axis of the brain and compare these with the well-described organization of adult neurogenic niches in *S. canicula*. To this end, we employed immunohistochemical techniques and in situ hybridization that enabled the localization of putative neurogenic niches. As already carried out by our group for *S. canicula* [37,38,46,47,48], we utilized antibodies against proliferating cell nuclear antigen (PCNA) as markers for proliferative cells, S100B as typical glial markers, Musashi-1 (Msi1) as markers for neuronal progenitors, and phospho-H3 (pH3) as mitotic markers. Additionally, we performed in situ hybridization for *Notch1*, *Notch3*, and *Sox2*, which are expressed by neural stem cells, as evidenced by studies performed in zebrafish. Active radial glial cells in zebrafish express both *Notch1* and *Notch3*, while quiescent radial glial cells express only *Notch3*; the deletion of *Notch1* affects cell division, while the removal of *Notch3* promotes proliferation [46,47,48]. We used the combination of *Notch1* and *Notch3* as markers for active stem cells, while the expression of *Notch3* alone served as a marker for quiescent stem cells. *Sox2* is known to universally label neural progenitors and stem cells throughout the central nervous system [49,50,51].

## 2. Results

**Figure 2:**
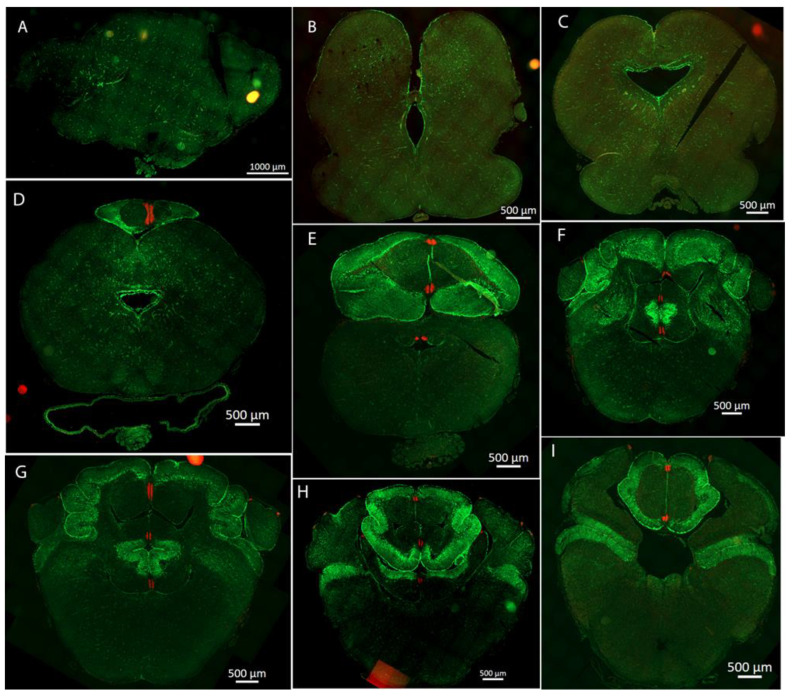
Representative map of the main neurogenic niches in the brain of *Raja asterias*, labeled for S100B (green) and PCNA (red). (**A**) telencephalon; (**B**) anterior mesencephalon and diencephalon; (**C**) medial mesencephalon; (**D**) posterior mesencephalon and anterior cerebellum; (**E**) cerebellum and posterior mesencephalon; (**F**–**I**) cerebellum, rhombencephalon, and cerebellar auricles.

To properly identify the localization of the main neurogenic niches of *R. asterias* and *T. ocellata,* we decided to conduct our analysis on coronal cryosections. A representative map of the main neurogenic niches present in *R. asterias* brain is reported in Figure 2. Additionally, we stained with PCNA and clarified and imaged a whole brain of R. asterias showing the three-dimensional localization of the areas of active cell proliferation (Supplementary Videos S1–S3).

### 2.1. Telencephalon

In *R. asterias*, we observed the presence of PCNA+/S100B+ cells throughout the entire length of the brain (Figure 2). S100B was used as a marker for glial cells, while PCNA was a marker for proliferative cells. This analysis revealed that the neurogenic niches are primarily located around the ventricles, as previously described for *S. canicula* [37,38]. We therefore proceeded with a more detailed analysis of this brain region.

**Figure 3:**
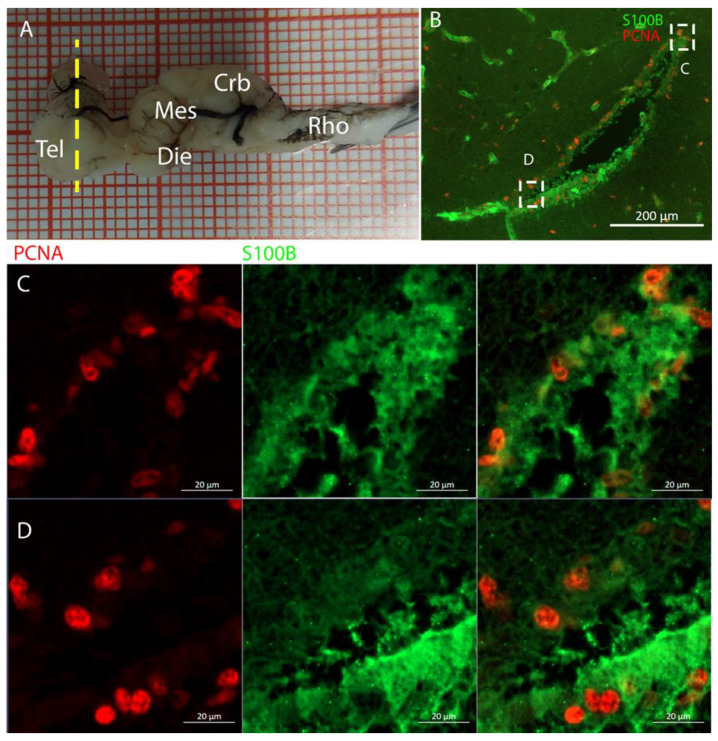
Localization of S100B (green)- and PCNA (red)-positive cells in the telencephalon of *Raja asterias*. (**A**) Brain of *R. asterias*; the yellow line indicates the rostro-caudal localization of the telencephalon. (**B**) Overview of the neurogenic niche in the telencephalon of *R. asterias*. (**C**) Magnification of the dorsal region of the telencephalic neurogenic niche. (**D**) Magnification of the ventral region of the telencephalic neurogenic niche.

In the rostral telencephalon, two ventricles situated on either side of the brain were recognized (Figure 3B). These ventricles caudally converged into a single large ventricle impar. The neurogenic niche was clearly identified by the presence of S100B+ cells that covered nearly all the surface of the ventricles; some of these glial cells were also PCNA+ (Figure 3B–D).

**Figure 4:**
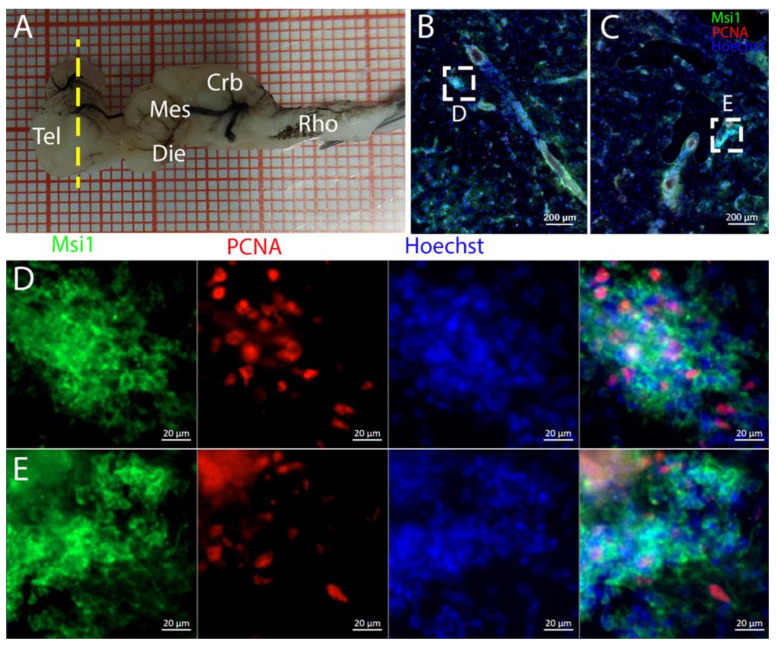
Localization of Msi1 (green)- and PCNA (red)-positive cells in the telencephalon of *Raja asterias*. (**A**) *R. asterias* brain; the yellow line indicates the rostro-caudal localization of telencephalon. (**B**) Overview of the left neurogenic niche in the telencephalon. (**C**) Overview of the right neurogenic niche in the telencephalon of *R. asterias*. (**D**) Magnification of the left neurogenic niche. (**E**) Magnification of the right neurogenic niche.

To characterize this neurogenic niche further, we performed immunostaining for Musashi-1 (Msi1), a general marker of neural stem cells, and phosphorylated histone H3 (pH3), which is a specific marker for the initial phases of mitosis. In this niche, Msi1+ cells encompassed the entire area of the ventricle, (Figure 4B), with numerous PCNA+ cells present within its expression domain (Figure 4C,D).

**Figure 5:**
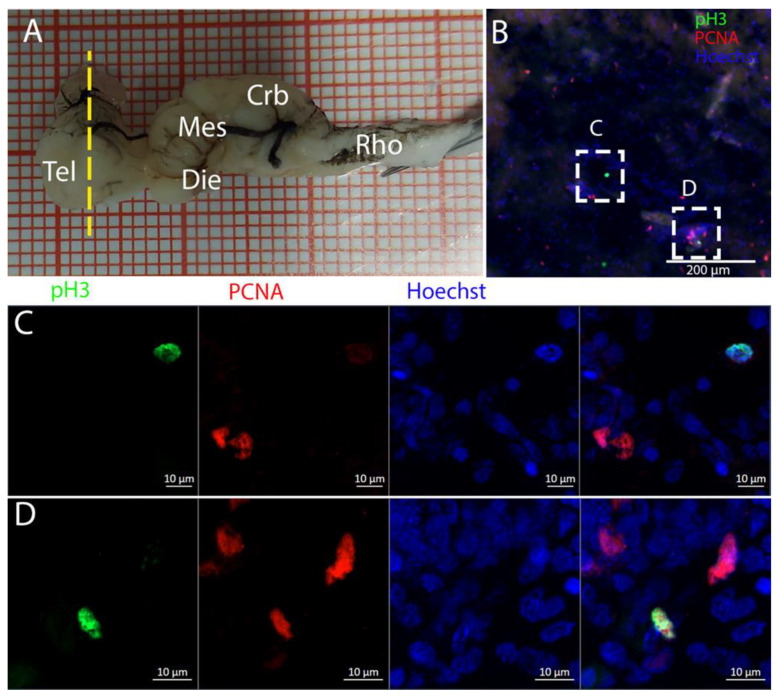
Localization of pH3 (green)- and PCNA (red)-positive cells in the telencephalon of *Raja asterias*. (**A**) *R. asterias* brain; the yellow line indicates the rostro-caudal localization of the telencephalon. (**B**) Overview of the neurogenic niche of the telencephalon. (**C**) Magnification of a pH3+ cell on the ventricular surface. (**D**) Magnification of a double-positive pH3+/PCNA cell in the periventricular area.

Cells resulting positive for pH3 and PCNA staining were observed throughout the ventricles, with some showing double labeling (Figure 5B), indicating that mitosis was occurring within this area (Figure 5C,D).

**Figure 6:**
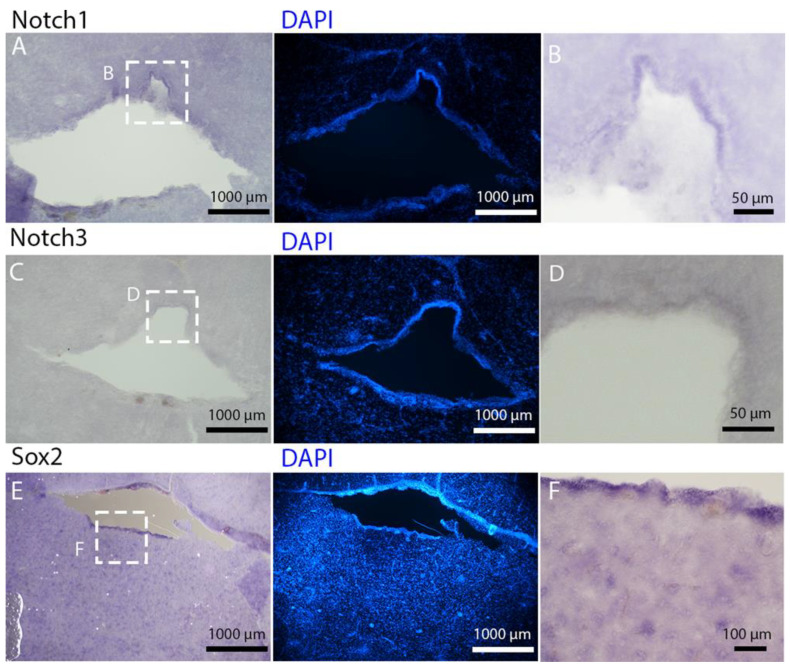
In situ hybridization for *Notch1*, *Notch3*, and *Sox2* on telencephalon of *Raja asterias*. (**A**) Overview of the telencephalic neurogenic niche stained for *Notch1*. (**B**) Magnification of *Notch1*+ area in the dorsal part of the neurogenic niche. (**C**) Overview of the telencephalic neurogenic niche stained for *Notch3*. (**D**) Magnification of *Notch3*+ area in the dorsal part of the neurogenic niche. (**E**) Overview of the telencephalic neurogenic niche stained for *Sox2*. (**F**) Magnification of *Sox2*+ area of picture (**E**).

To deepen the molecular characterization of this niche, we cloned the orthologs of *Notch1*, *Notch3*, and *Sox2* and visualized their expression domains using in situ hybridization (Figure 6A,F). The expression domains of these canonical neural stem cell markers further confirmed the neurogenic nature of the niche surrounding the lateral ventricles.

*Notch1* and *Notch3* expression was subtle, restricted to a narrow band in the dorsal region of the posterior telencephalic ventricle. At this level, the ventricles had already merged into a single one. The dorsal part of this area exhibited a similar morphology to the dorsal area of the mesencephalic ventricle (see Section 2.2), presenting a higher density of cells.

In contrast, *Sox2* expression was broadly distributed along the entire ventricular wall (Figure 6E,F). A notable characteristic of *Sox2* labeling is its presence in numerous cells and within the parenchyma across the entire telencephalon surface (Figure 6E,F). All three RNAs were expressed in the region we identified as the neurogenic niche, as clearly evidenced by the comparison with the staining for S100 and PCNA in a section adjacent to the ones selected for in situ hybridization (Figure S3A–D).

**Figure 7:**
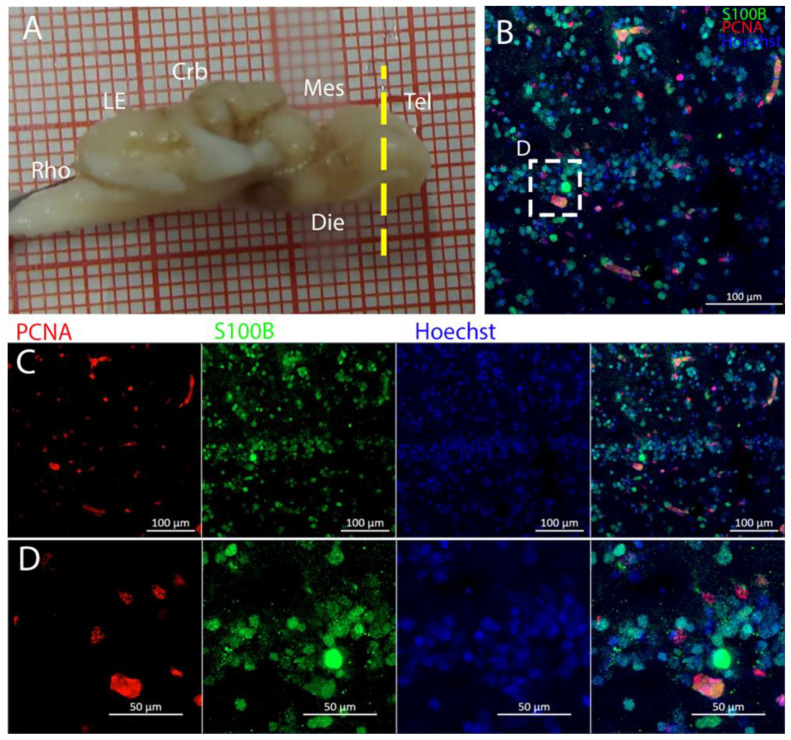
Localization of S100B (green)- and PCNA (red)-positive cells in the telencephalon of *Torpedo ocellata*. (**A**) *T. ocellata* brain; the yellow line indicates the rostro-caudal localization of the telencephalon. (**B**) Neurogenic niche in the telencephalon of *T. ocellata*. (**C**) Single channels of the neurogenic niche in the telencephalon of *T. ocellata*. (**D**) Magnification of S100B+ and PCNA+ cells.

In *T. ocellata*, the telencephalon exhibited a similar symmetrical bilateral anatomy. In the two lateral ventricles, S100B+ cells were tightly distributed around the ventricular wall, but they were also abundant in the parenchyma (Figure 7). PCNA+ cells were predominantly, but not exclusively, distributed around the ventricles. Many PCNA+ cells appeared to be also S100B+, but showing a weaker signal intensity of the glial marker (Figure 7D).

**Figure 8:**
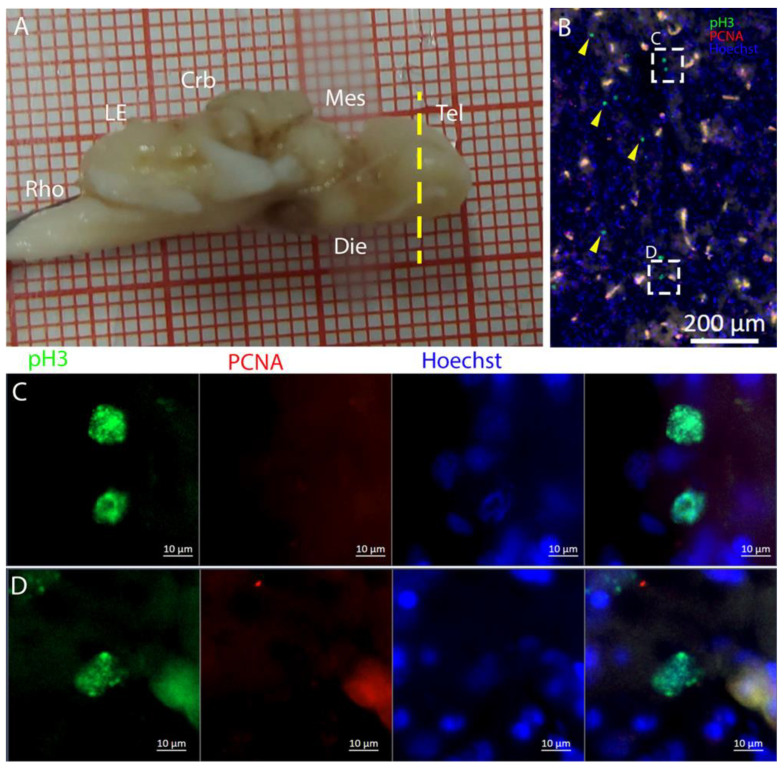
Localization of pH3 (green)- and PCNA (red)-positive cells in the telencephalon of *T. ocellata*. (**A**) *T. ocellata* brain; the yellow line indicates the rostro-caudal localization of the telencephalon. (**B**) Overview of the neurogenic niche in the telencephalon of *T. ocellata*. Yellow arrows indicate pH3+ cells in the parenchyma. (**C**) Magnification of the dorsal part of the telencephalic neurogenic niche. (**D**) Magnification of the ventral part of the telencephalic neurogenic niche.

Along the surface of the ventricle, a few pH3+ cells were visible (Figure 8B), as shown in the magnifications (Figure 8C,D). The presence of pH3+ cells could be observed also in the parenchyma (Figure 8B, yellow arrows). Notably, all the pH3+-positive cells appeared to be PCNA- (Figure 8C,D) in this species.

### 2.2. Mesencephalon/Optic Tectum

In *R. asterias,* the telencephalic ventricles extended caudally and merged medially into a single diencephalic opening that continued to the mesencephalic and rhombencephalic areas.

**Figure 9:**
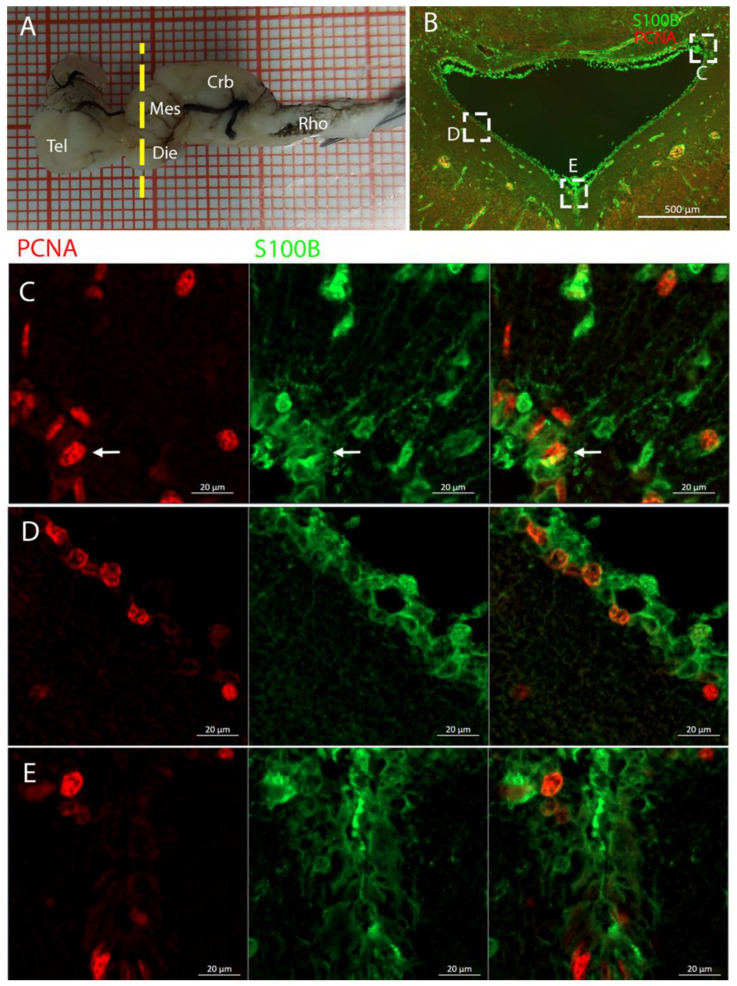
Localization of S100B (green)- and PCNA (red)-positive cells in the mesencephalon of *Raja asterias*. White arrow indicates S100B+/PCNA+ cells. (**A**) *R. asterias* brain; the yellow line indicates the rostro-caudal localization of the mesencephalon. (**B**) Overview of the neurogenic niche in the mesencephalon of *R. asterias*. (**C**) Magnification of the dorsal part of the mesencephalic neurogenic niche. (**D**) Magnification of the median part of the mesencephalic neurogenic niche. (**E**) Magnification of the ventral part of the mesencephalic neurogenic niche.

Staining for PCNA/S100B in the mesencephalon clearly highlighted the peri-ventricular localization of the niche (Figure 9B). Following the classical RG structure, cell bodies were located around the ventricular area, facing the lumen, while the processes extended through the parenchyma (Figure 9C). Some of these cells were S100B+/PCNA+, indicating active RG cells (Figure 9C–E). Additionally, we verified the presence of postmitotic neurons adjacent to the mesencephalic neurogenic niche by staining cells expressing the enzyme Tyrosine Hydroxylase (TH, Figure S2). Mature neurons were distributed in the parenchima and some cell bodies were located in close proximity to the ventricular wall where the neurogenic niche was located (Figure S2C).

**Figure 10:**
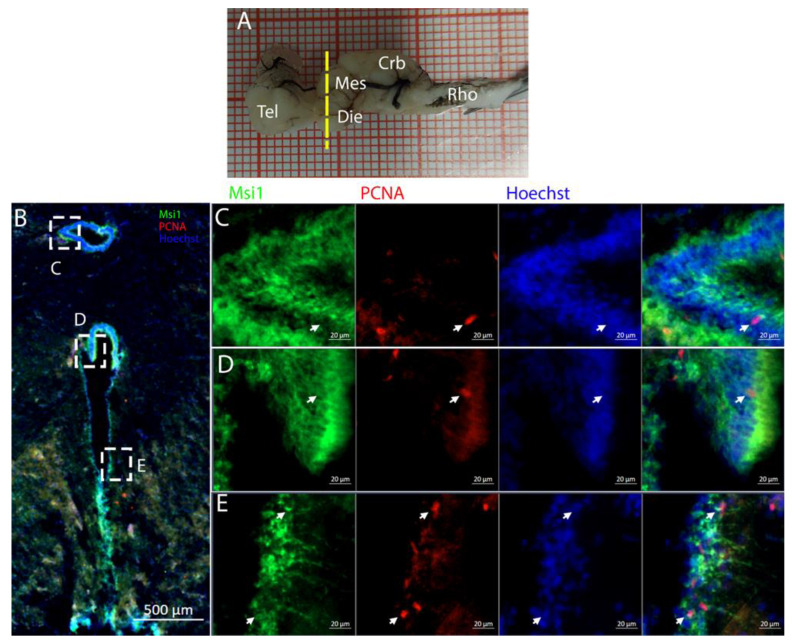
Localization of Msi1 (green)- and PCNA (red)-positive cells in the mesencephalon of *Raja asterias*. White arrows indicate Msi1+/PCNA+ cells. (**A**) *R. asterias* brain; the yellow line indicates the rostro-caudal localization of mesencephalon. (**B**) Overview of the neurogenic niche in the mesencephalon of *R. asterias*. (**C**) Magnification of the dorsal part of the mesencephalic neurogenic niche. (**D**) Magnification of the median part of the mesencephalic neurogenic niche. (**E**) Magnification of the ventral part of the mesencephalic neurogenic niche.

At this level, the ventricle was anatomically divided into a dorsal and a ventral part (Figure 10B–D squared areas), which merged into a single ventricular cavity more posteriorly (as visible in the following figures). The dorsal part of the ventricle (Figure 2B) was characterized by a zone of high cell density. This region was absent caudally, where the ventricle changed position and shape, becoming larger. (Figure 2C). In the high-cell-density area, PCNA+ cells were noticeably more dense, although the S100B signal was almost absent (Figure S1). Cells located in this area were positive for Msi1 (Figure 10B) and showed no evidence of processes radiating in the parenchyma (Figure 10C,D). This indicated their identity as being distinct from that of RG, suggesting a neuroepithelial nature. In the lateral and ventral part of the ventricle, cells showed a more distinct RG morphology (Figure 10E).

**Figure 11:**
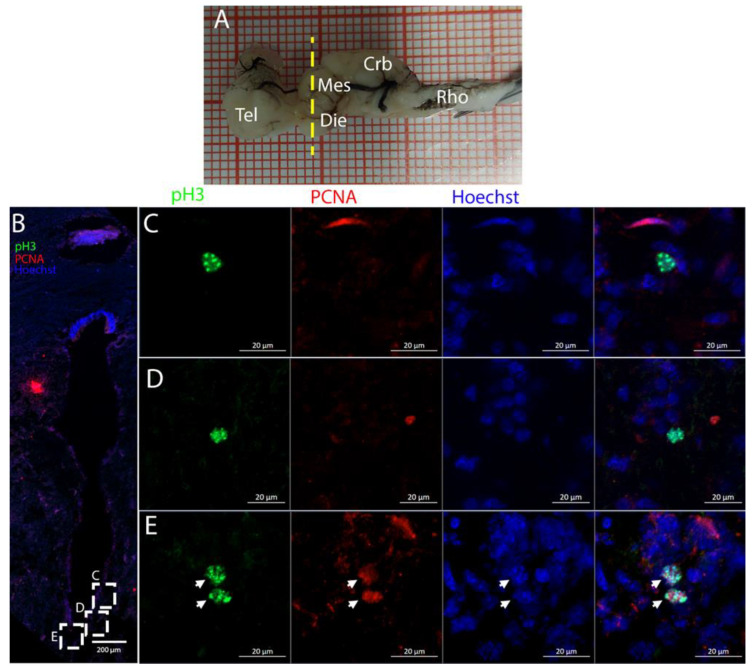
Localization of pH3 (green)- and PCNA (red)-positive cells in the mesencephalon of *Raja asterias*. White arrows indicate pH3+/PCNA+ cells. (**A**) *R. asterias* brain; the yellow line indicates the rostro-caudal localization of the mesencephalon. (**B**) Overview of the neurogenic niche in the mesencephalon of *R. asterias*. (**C**) Magnification of a pH3+ cell in the ventral part of the mesencephalic neurogenic niche. (**D**) Magnification of a pH3+ cell in the ventral part of the mesencephalic neurogenic niche. (**E**) Magnification of two double-positive pH3+/PCNA+ cells in the ventral part of the mesencephalic neurogenic niche.

Staining for pH3/PCNA in this area (Figure 11) revealed the presence of actively dividing cells, occasionally showing coexpression with PCNA, especially in the ventral part of the ventricle (Figure 11E, white arrows). The pH3+ cells shown in Figure 11E were located in close proximity to one another, representing a classic example of a recent division event.

**Figure 12:**
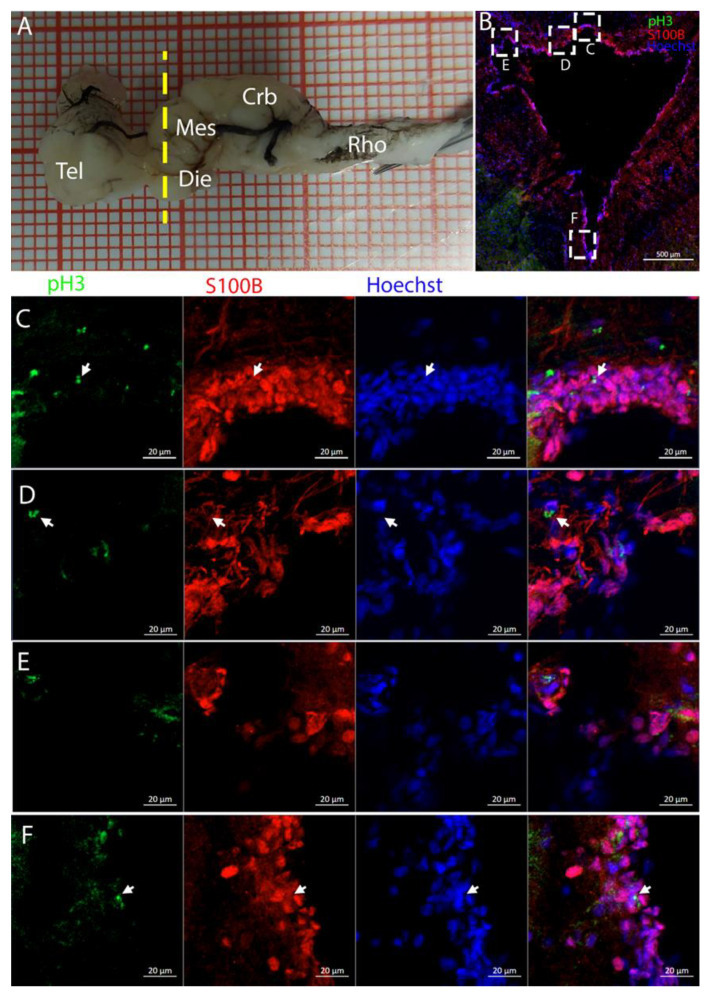
Localization of pH3 (green)- and S100B (red)-positive cells in the mesencephalon of *Raja asterias*. White arrows indicate pH3+/S100B+ cells. (**A**) *R. asterias* brain; the yellow line indicates the rostro-caudal localization of the mesencephalon. (**B**) Overview of the neurogenic niche in the mesencephalon of *R. asterias*. (**C**) Magnification of the dorso-median part of the mesencephalic neurogenic niche. (**D**) Magnification of the dorso-median part of the mesencephalic neurogenic niche. (**E**) Magnification of the dorso-lateral part of the mesencephalic neurogenic niche. (**F**) Magnification of the ventral part of the mesencephalic neurogenic niche.

Labeling for pH3/S100B confirmed the presence of a few double-positive cells along the ventricle walls (Figure 12C–F, white arrows).

**Figure 13:**
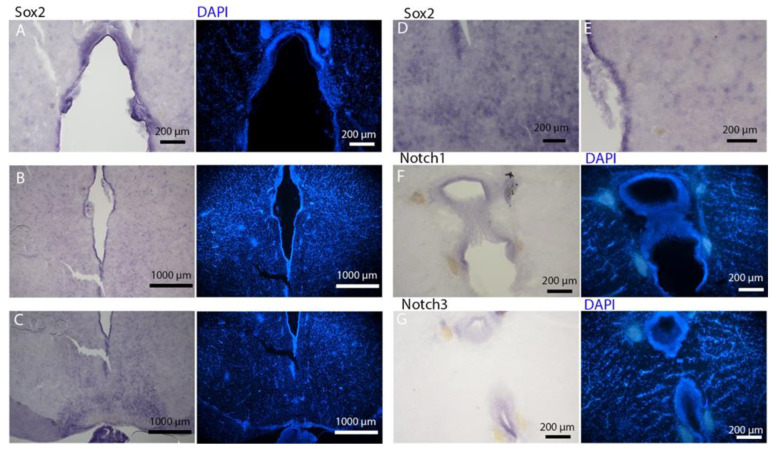
In situ hybridization for *Notch1*, *Notch3*, and *Sox2* on the mesencephalon of *Raja asterias*. (**A**) Dorsal part of the mesencephalic neurogenic niche stained for *Sox2*. (**B**) Median part of the mesencephalic neurogenic niche stained for *Sox2*. (**C**) Ventral part of the mesencephalic neurogenic niche stained for *Sox2*. (**D**) Magnification of the ventral part of mesencephalic neurogenic niche. (**E**) Magnification of median part of the mesencephalic neurogenic niche stained for *Sox2*. (**F**) Mesencephalic neurogenic niche stained for *Notch1*. (**G**) Mesencephalic neurogenic niche stained for *Notch3*.

In situ hybridization for *Notch1*, *Notch3*, and *Sox2*, showed the expression of these genes in the dorsal area of the niche characterized by a high-density cellular population (Figure 13A,F,G). Signal intensity decreased ventrally, especially for *Notch1* (Figure 13F). The *Sox2* signal remained visible along all the ventricular surfaces as well as in the parenchyma, showing a more extensive area of expression compared to *Notch1* and *Notch3* (Figure 13B,C). *Sox2* expression in the parenchyma was widespread and punctate, similar to that observed in the telencephalon (Figure 13B–E). In the most ventral area of the section (Figure 13C), we could recognize an arc-shaped distribution of densely packed *Sox2+* cells.

As in the telencephalon, the three genes associated with adult neurogenesis were expressed in the area that we identified as the neurogenic niche from a comparison of similar sections stained for both Ms1 and S100B (Figure S3E–I).

**Figure 14:**
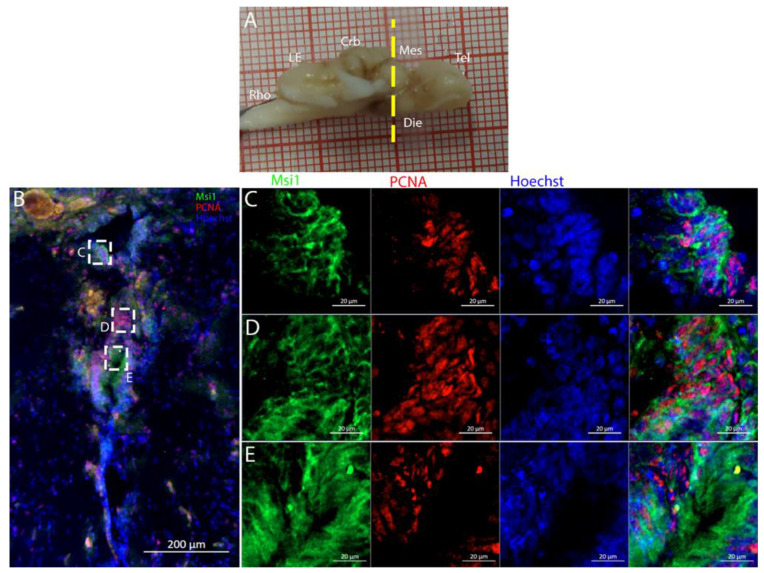
Localization of Msi1 (green)- and PCNA (red)-positive cells in the mesencephalon of *Torpedo ocellata*. (**A**) *T. ocellata* brain; the yellow line indicates the rostro-caudal localization of the mesencephalon. (**B**) Overview of the neurogenic niche in the mesencephalon of *T. ocellata*. (**C**) Magnification of the dorsal part of the mesencephalic neurogenic niche. (**D**) Magnification of the median part of the mesencephalic neurogenic niche. (**E**) Magnification of the median part of the mesencephalic neurogenic niche.

The distribution of the neurogenic niche of the mesencephalon in *T. ocellata* (Figure 14) appeared to be similar to that of *R. asterias* (Figure 10). The analyzed area in *T. ocellata* was located in a slightly posterior area of the brain with respect to the one analyzed in *R. asterias*. This led to the appearance of one single ventricle in *T. ocellata* derived from the fusion of the most anterior separated cavities, visualized as dorsal and ventral ventricles in Figure 10.

The cells located around the ventricle were Msi1+ (Figure 14B–E), showing the strongest signal mainly in the medial part of the ventricle. In this region, a large number of PCNA+ cells were also present.

**Figure 15:**
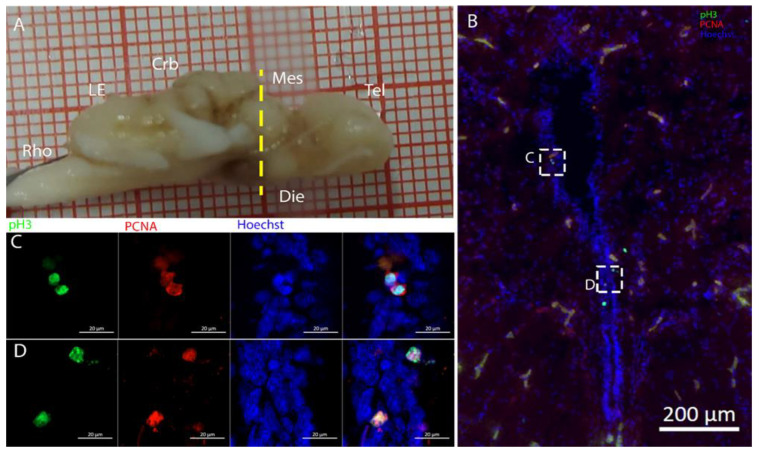
Localization of pH3 (green)- and PCNA (red)-positive cells in the mesencephalon of *Torpedo ocellata*. (**A**) *T. ocellata* brain; the yellow line indicates the rostro-caudal localization of the mesencephalon. (**B**) Overview of the neurogenic niche in the mesencephalon of *T. ocellata*. (**C**) Magnification of the median part of the mesencephalic neurogenic niche. (**D**) Magnification of the ventral part of the mesencephalic neurogenic niche.

Also in this area, it was possible to identify a few PCNA+/pH3+ cells, mainly visible in the medial and ventral area of the mesencephalic cavity (Figure 15C,D).

### 2.3. Cerebellum

**Figure 16:**
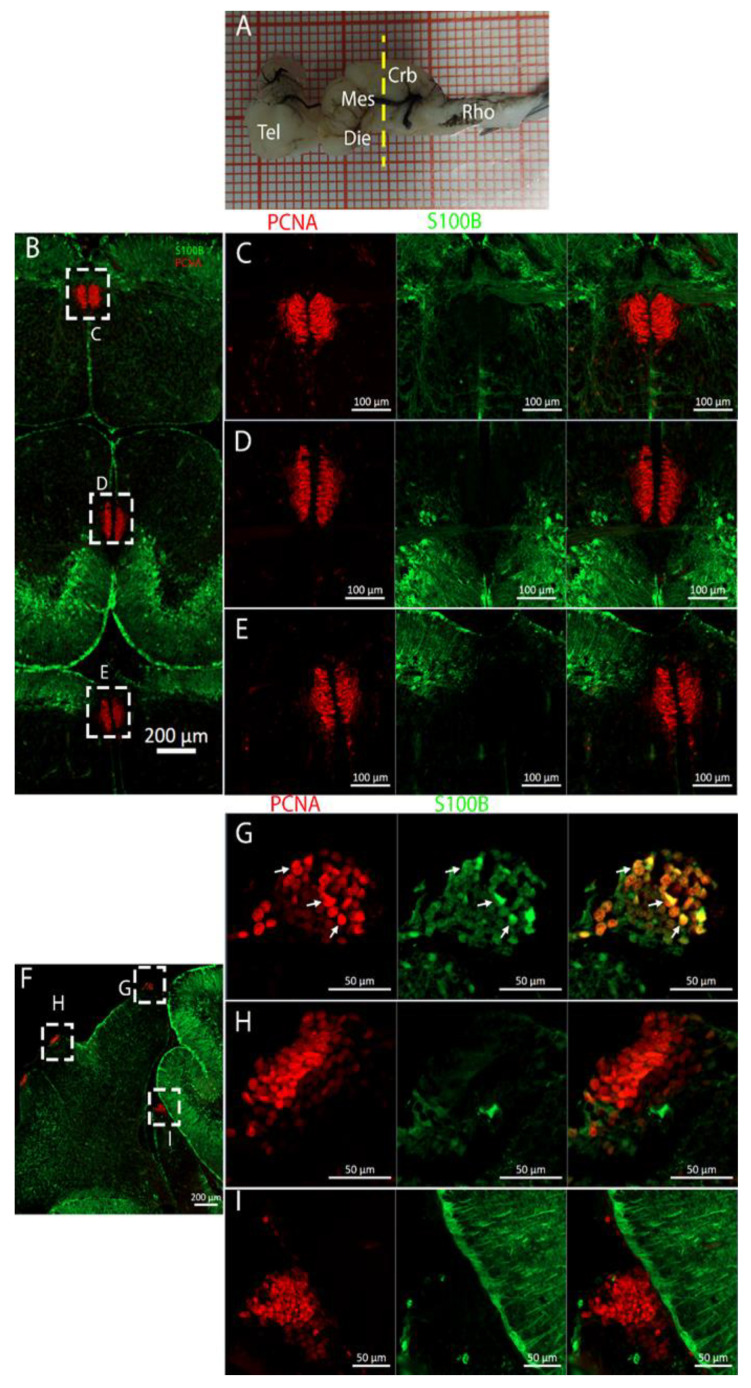
Localization of S100B (green)- and PCNA (red)-positive cells in the cerebellum of *Raja asterias*. (**A**) *R. asterias* brain; the yellow line indicates the rostro-caudal localization of the cerebellum. (**B**) Overview of the neurogenic niches in the central part of the cerebellum of *R. asterias*. (**C**) Magnification of the dorsal cerebellar neurogenic niche. (**D**) Magnification of the ventral cerebellar neurogenic niche. (**E**) Magnification of the MON niche. (**F**) Overview of the neurogenic niches in the lateral part of the cerebellum of *R. asterias*. (**G**) Magnification of the dorsal auricular neurogenic niche. White arrows indicate double-positive PCNA+/S100B+ cells. (**H**) Magnification of the ventral auricular neurogenic niche. (**I**) Magnification of the DON niche.

The *Raja asterias* cerebellar area stained for PCNA and S100B presented different restricted but well-defined areas of neurogenesis composed of tightly packed cells (Figure 16). We identified three different niches in the medial region (Figure 16B) and four in the lateral area (Figure 16F). Among these seven different niches, two were located in the *Corpus Cerebelli* (Figure 16C,D), one was in the area of the medial and dorsal *Octavolateralis Nuclei* (MON and DON, Figure 16E), and two cerebellar-like nuclei were located ventrally to the cerebellum and used to receive sensory stimuli from the lateral line system. The remaining four niches were located in the cerebellar auricles (Figure 16F–I), two paired lobes functionally associated with the lateral line centers, also with cerebellar-like structure. The only niche formed by cells coexpressing PCNA and S100B was located dorsally in the dorsal auricles (Figure 16G). All the other niches (Figure 16C–E,H,I) presented mutually exclusive staining for PCNA and S100B, similar to what has been described for *Scyliorhinus canicula* [38].

**Figure 17:**
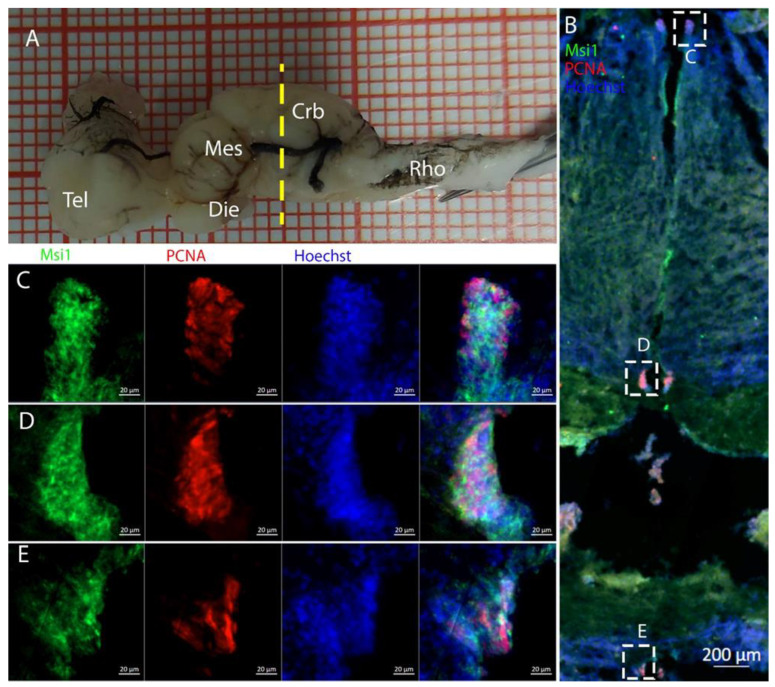
Localization of Msi1 (green)- and PCNA (red)-positive cells in the cerebellum of *Raja asterias*. (**A**) *R. asterias* brain; the yellow line indicates the rostro-caudal localization of the cerebellum. (**B**) Overview of the neurogenic niches in the cerebellum of *R. asterias*. (**C**) Magnification of the dorsal cerebellar neurogenic niche. (**D**) Magnification of the ventral cerebellar neurogenic niche. (**E**) Magnification of the MON niche.

Niches located along the medial line of the cerebellum were positively labeled for Msi1 (Figure 17): all PCNA+ cells also appeared to be Msi1+.

**Figure 18:**
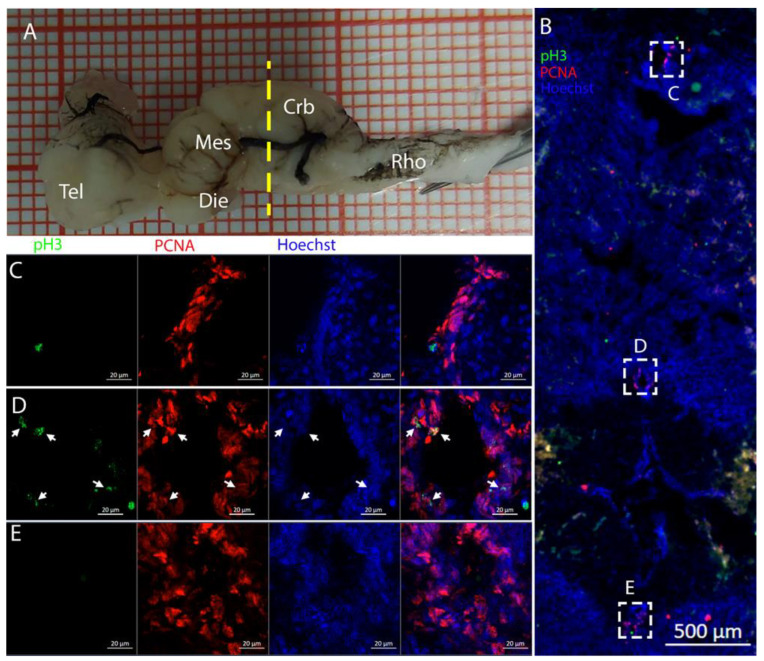
Localization of pH3 (green)- and PCNA (red)-positive cells in the cerebellum of *Raja asterias*. (**A**) *R. asterias* brain; the yellow line indicates the rostro-caudal localization of the cerebellum. (**B**) Overview of the neurogenic niches in the cerebellum of *R. asterias*. (**C**) Magnification of the dorsal cerebellar neurogenic niche. (**D**) Magnification of the ventral cerebellar neurogenic niche. White arrows indicate double-positive PCNA+/pH3+ cells. (**E**) Magnification of the MON niche.

We found evidence of actively dividing cells both in the ventral cerebellar and DON niches by identifying double-positive cells for PCNA and pH3 (Figure 18E). We did not find any S100B+/pH3+ cells at the level of the cerebellum.

**Figure 19:**
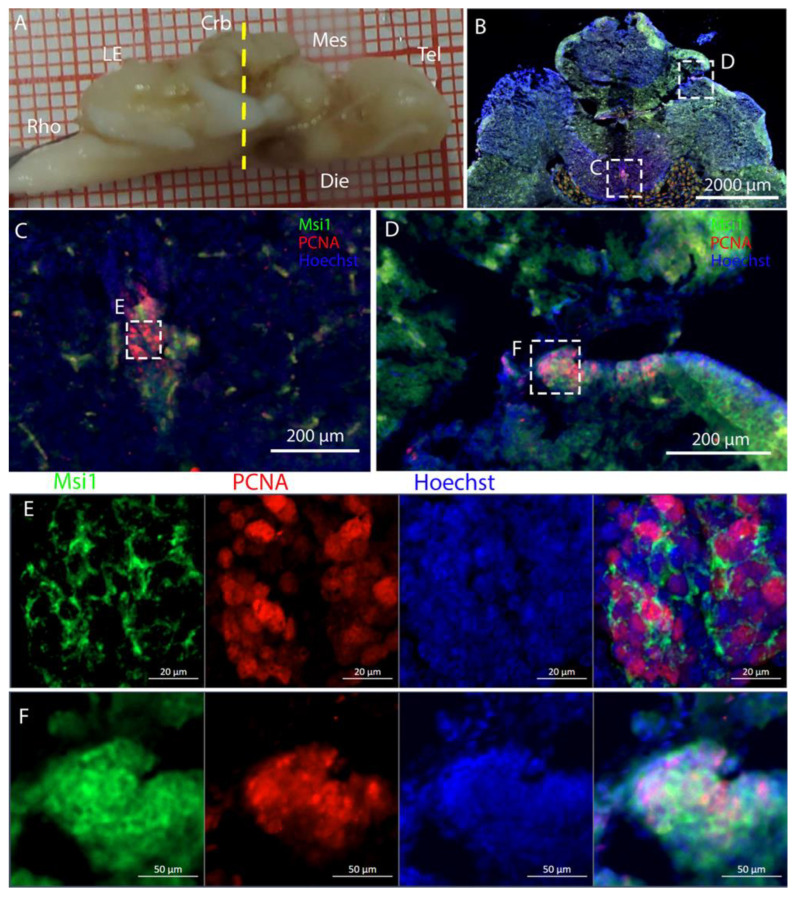
Localization of Msi1 (green)- and PCNA (red)-positive cells in the cerebellum of *T. ocellata*. (**A**) *T. ocellata* brain; the yellow line indicates the rostro-caudal localization of the cerebellum. (**B**) Overview of the cerebellum of *T. ocellata*. (**C**) Overview of the MON neurogenic niche. (**D**) Overview of the neurogenic niche of the right auricle. (**E**) Magnification of the MON neurogenic niche. (**F**) Magnification of the neurogenic niche of the right auricle.

**Figure 20:**
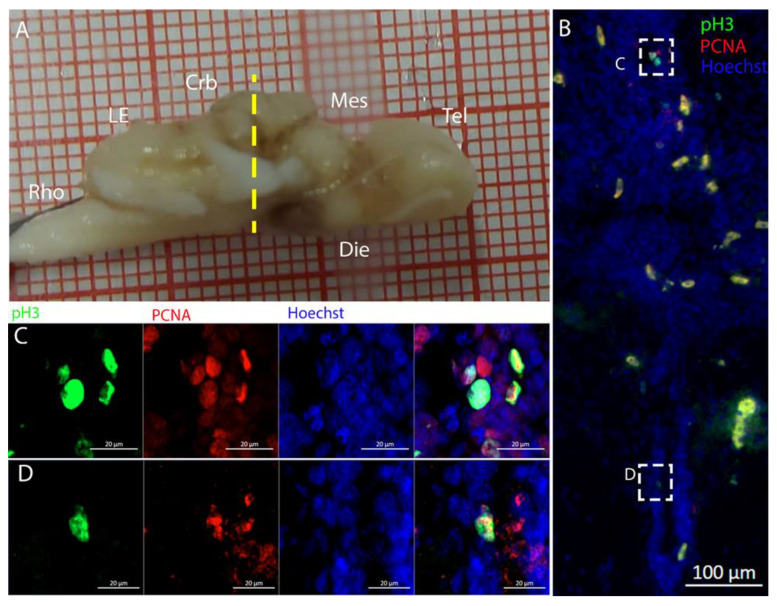
Localization of pH3 (green)- and PCNA (red)-positive cells in the cerebellum of *Torpedo ocellata*. (**A**) *T. ocellata* brain; the yellow line indicates the rostro-caudal localization of the cerebellum. (**B**) Overview of the neurogenic niches in the cerebellum of *T. ocellata*. (**C**) Magnification of the dorsal part of the cerebellar neurogenic niche. (**D**) Magnification of the ventral part of the cerebellar neurogenic niche.

Contrary to what we observed in *R. asterias,* in the most anterior part of the cerebellar structure of *T. ocellata*, we could identify only the DON niche, but not the *Corpus Cerebelli* niches (Figure 19B,C,E). This niche harbored PCNA+/Msi1+ cells, resembling the morphology of the niches observed in *R. asterias* for both density and shape (Figure 19C,E). At this level, we also identified auricular niches positive for PCNA and Msi1 (Figure 19D,F). More posteriorly, in addition to the DON niche, a ventral cerebellar niche, presenting positive staining for PCNA and pH3, became visible (Figure 20B,C). Both niches showed a fainter PCNA signal than what was observed in *R. asterias*.

**Figure 21:**
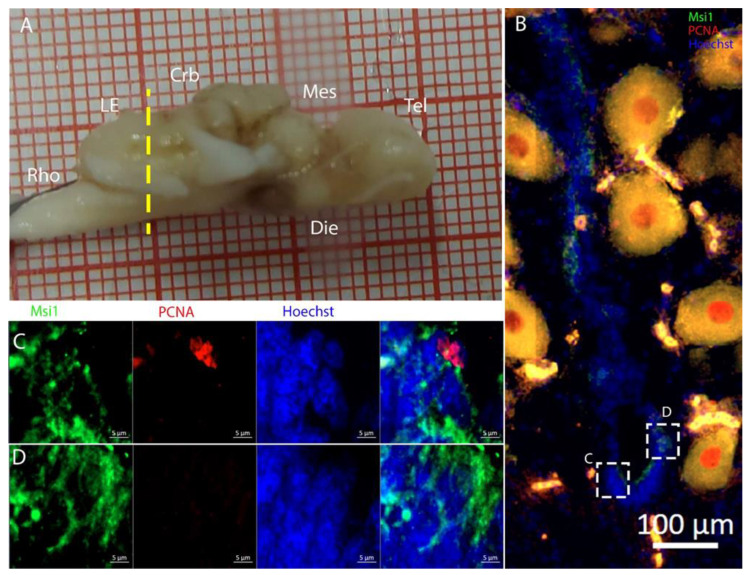
Localization of Msi1 (green)- and PCNA (red)-positive cells in the *Lobus Electricus* (LE) of *Torpedo ocellata*. (**A**) *T. ocellata* brain; the yellow line indicates the rostro-caudal localization of the LE. (**B**) Overview of the neurogenic niche in the *LE* of *T. ocellata*. (**C**) Magnification of the ventral part of the LE neurogenic niche. (**D**) Magnification of the ventral part of the LE neurogenic niche.

**Figure 22:**
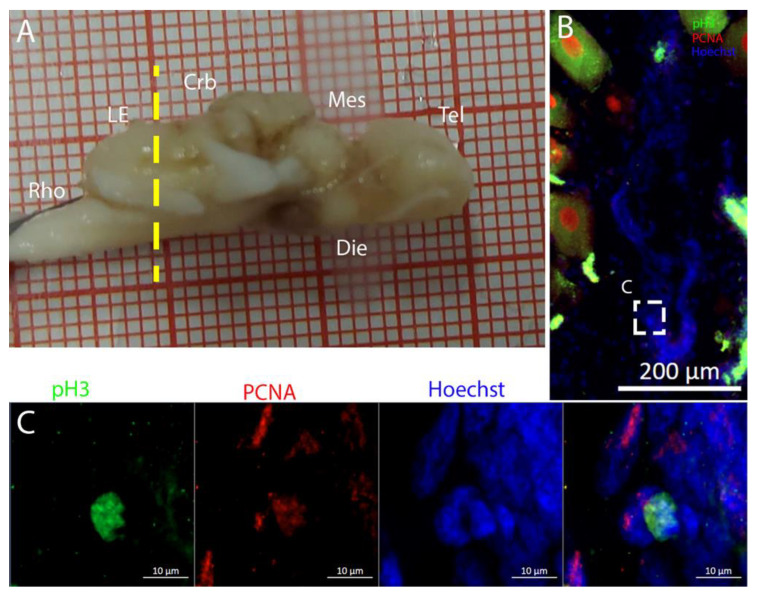
Localization of pH3 (green)- and PCNA (red)-positive cells in the *Lobus Electricus* (LE) of *Torpedo ocellata*. (**A**) *T. ocellata* brain; the yellow line indicates the rostro-caudal localization of the LE. (**B**) Overview of the neurogenic niche in the LE of *T. ocellata*. (**C**) Magnification of the ventral part of the LE neurogenic niche.

We found the presence of neurogenic cells in the *Lobus Electricus* (LE) of *T. ocellata*, located posteriorly to the cerebellum (Figure 21A). At this level, the ventricle was very narrow and surrounded by giant cells (Figure 21B). Labeling this region for Msi1, PCNA, and pH3, we identified an area containing progenitor cells along the most ventral part of the ventricle (Figure 21B–D). In this area, there was a scarce presence of PCNA+ cells (Figure 21C,D). Even more rare were pH3+ cells (Figure 22C).

In the following table (Table 1), we summarize the staining observed in the different brain areas of *R. asterias* and *T. ocellata*.

## 3. Discussion

The aim of this study was to increase our knowledge about the phenomenon of adult neurogenesis in the chondrichthyans group, with particular attention to the *Batoidea* group, to understand whether widespread neurogenesis could represent an ancestral trait and how neurogenic niches are shaped in response to morphogenic variations across brain macrostructure. In particular, rays were characterized by a comparatively larger telencephalon and much smaller ventricle surface as well as a larger and more folded cerebellum. *T. ocellata* on the other hand was characterized by the *Lobus Electricus*, a structure that innervates its electric organ.

### 3.1. Telencephalon

The telencephalon of both *R. asterias* and *T. ocellata* presented two symmetrical ventricles that appeared partially collapsed, with a reduced luminal cavity. In both species, we found the presence of neurogenic markers along the ventricular surface throughout the rostro-caudal axis, presenting positive signals for both S100B and Msi1. These cells showed very clear processes that extended from the ventricle to the parenchyma, an indication of their RG nature. To confirm the presence of actively dividing cells, we also performed staining for pH3. The presence of pH3+ cells confirmed that mitotic events occurred around the ventricles. Actively dividing cells were also present in the parenchyma, but their nature was not investigated. They could represent transit-amplifying neural progenitors, glial progenitors, or oligodendrocyte progenitors. It should be noted that the density of S100B cells in the parenchyma was much higher than in *S. canicula* and S100B+ cells in rays, which have been suggested to have an astrocyte-like morphology [50,51]. It is possible that these astrocytes retain the possibility of dividing in the adult brain of rays, explaining the high density of dividing cells in the parenchyma. To further characterize the neurogenic niche in *R. asterias*, we performed in situ hybridizations for *Notch1*, *Notch3*, and *Sox2*. Probes were designed based on homology since, at the time, no genomic data were available for *R. asterias*. We thus used reference sequences from genes of the phylogenetically close species, *Amblyraja radiata*. *Notch1* and *Notch3* revealed a patterning of the telencephalic ventricles as they were expressed only in its dorsal part, in a spatially limited region (Figure 6A–D), and no signal from the remaining part of the ventricle was detectable. The coexpression of *Notch1* and *Notch3* is commonly linked to active neurogenic stem cells and is present in very early precursors [44,45,46]. The *Sox2* signal is also linked to neurogenic stem cells but is only expressed in RG and immature neuronal progenitors [47,48,49]. The expression of *Sox2* was more distributed alongside the surface of the ventricle, confirming the stem cell nature of the cells in the subventricular zone. Together, these data indicate a difference between a more dorsal area of primordial cells expressing *Notch1* and *Notch3* and a distribution of different types of stem cells around the rest of the ventricle. *Sox2* was also detectable and abundant in the parenchyma, in line with the observations of Docampo-Seara et al. [37], who detected *Sox2+* cells in the telencephalic parenchyma of *S. canicula*. These *Sox2+* cells appear to be more abundant in rays and may represent transit progenitors or a specialized population of astrocytes. Further investigations are needed to characterize the nature of these cells. The nature of the neurogenic niche appears to be different in *T. ocellata*. S100B+ cells did not present the typical shape of RG but appeared to be spherical, with no prolonged processes apart from in rare cases (Figure 7C). There was an abundant presence of S100B+ cells along the ventricle and into the parenchyma, but only a few of those appeared to be also PCNA+. This may indicate a limited presence of actively dividing progenitors, confirmed by the limited number of pH3+ cells (Figure 8B). As discussed for *R. asterias,* the presence of pH3+ cells (both positive and negative for PCNA staining) in the parenchyma could indicate their identity as transient amplifying progenitors (TAP) or glial or oligodendrocyte progenitors. To better understand the nature of the parenchymal PCNA+ cells and their distribution, we plan to repeat and improve this characterization.

### 3.2. Mesencephalon

The optic tectum is a large and prominent region in this species. Unlike mammals, fish have evolved this area more extensively than the telencephalon, probably reflecting their adaptation to their ecological niche [52]. In the mesencephalon, the situation of both species appears to be more similar: at this level, there is a big central ventricle surrounded by S100B+ cells with numerous PCNA+ cells (Figure 9, Figure 12 and Figure 14). In the dorsal area in the *R. asterias* dorsal ventricle, there was a region of high-density cells that were S100B-. We could clearly see how S100B+ cells localized all around the ventricle except for this area. Despite the absence of S100B+ cells, there were numerous PCNA+ cells, more than in the rest of the ventricular surface. These dorsally located cells were strongly Msi1+ (Figure 10), clearly indicating their neurogenic nature. These cells were the only cells of the niche expressing both *Notch1* and *Notch3*. These cells were also positive for *Sox2+*, together with the other cells localized on the rest of the ventricular surface. Considering the differential labeling of this area, the rest of the ventricular surface, and the parenchyma, we hypothesize that there is a main area of neurogenesis, sustained by a cell type different than RGs, probably made by neuroepithelial cells, as observed in *S. canicula* in [38]. This observation is sustained by the presence of S100B-/PCNA+ cells. The remaining part of the ventricle is probably composed of RGs, with them being S100B+ and positive for other markers like *Sox2* and Msi1 but negative for *Notch1* and *Notch3*. Some of these cells are active and probably divide with asymmetric divisions, generating immature neuronal precursors that are visible as *Sox2*+ or PCNA+ cells in the parenchyma. The mesencephalic niche seemed to have mixed features of a neuroepithelial niche and a radial glia. Several cells were also stained for the marker of division pH3, as observed in the ventral mesencephalon (Figure 11). In particular, the presence of two recently divided cells (Figure 11E, white arrows) suggested a fast turnover of this proliferative cell population. The situation in *T. ocellata* was different since we were not able to identify a region with densely packed cells. All the ventricular surfaces appeared to be homogeneous, with double-positive S100B+/PCNA+ cells and Msi1+ cells distributed all around the ventricle. The main difference from the *R. asterias* mesencephalic ventricle was the absence of strong S100B positivity in all the ventricular cells: while all cells appeared to be Msi1+, not all of them showed the expression of S100B. This indicated that the niche was composed of a mixed population of RGs and other types of progenitor cells.

### 3.3. Cerebellum

The cerebellum of *Raja asterias* anteriorly exhibited seven distinct neurogenic niches, with three located in the medial region and four in the lateral area. Notably, two of these niches were situated in the *Corpus Cerebelli*, one was in the medial and dorsal *Octavolateralis Nuclei* (MON and DON), and four were in the cerebellar auricles (Figure 16). The identification of multiple neurogenic niches in both medial and lateral regions of the cerebellum, including those in the *Corpus Cerebelli* and cerebellar auricles, underscores the complexity of the neural development of this species in these areas. The only niche where cells co-expressed PCNA and S100B was located dorsally in the dorsal auricles (Figure 16G), while all the other cerebellar niches showed mutually exclusive staining for these markers, similar to what has been described for *Scyliorhinus canicula* [38]. These S100+/PCNA- cells resembled the morphology of the Bergmann glia, a type of radial astrocyte found in the human cerebellar cortex: this similarity is consistent with previous studies highlighting the role of Bergmann glia in guiding neuronal migration and supporting neural development in the cerebellum [53,54]. All PCNA+ cells in the medial line of the cerebellum were also Msi1+, indicating their role as neural progenitors and their putative neuroepithelial origin (Figure 17). Moreover, the expression of Msi1 in niches along the medial line of the cerebellum, particularly in PCNA+ cells, indicated that Msi1 may play a role in identifying and maintaining neural progenitor cells within these regions, as observed in other chondrichthyans species [38]. The absence of S100B staining and a wall-like, shaped, densely packed distribution of these cells reinforce the idea of their possible neuroepithelial origin. Msi1 is widely recognized as a marker for neural progenitor cells, including neuroepithelial stem cells [55,56,57]. However, further investigation with additional neuroepithelial and epithelial markers—not available in our laboratory—would be necessary to confirm this observation. More posteriorly, the number of cerebellar niches increased to nine, including the bilateral auricular niches, not detectable more rostrally. The auricular niches appeared to be similar in nature to the cerebellar ones, showing the classic, mutually exclusive labeling of PCNA and S100B (Figure 16G). We were not able to apply the in situ hybridization experiment to the cerebellum slices, so we plan to do this in the future. The presence of double-positive cells for PCNA and pH3 in the ventral cerebellar and DON niches suggests active cell division and neurogenesis in these areas. However, the absence of S100B+/pH3+ cells in the cerebellum indicated that differentiated cells may not have been actively dividing, consistent with their role in supporting neural function rather than proliferation (Figure 18). For technical reasons, we could not observe S100B+ cells in the cerebellum of *T. ocellata*. and plan to collect these data in the future. We did collect evidence of labeling for Msi1+, PCNA+, and pH3+. At this level, the ventricular structure became smaller but kept the same characteristics as before. Cells around the ventricle were Msi1+, with a high concentration of PCNA+ cells (Figure 19) and pH3+ cells (Figure 20), which demonstrated how, even if the niche was smaller, it was still perfectly active. Comparing the cerebellar structure of *R. asterias* and *T. ocellata*, we observed differences in the distribution and number of neurogenic niches. While *R. asterias* exhibited multiple niches in both the *corpus cerebelli* and cerebellar auricles, *T. ocellata* showed a more restricted pattern, with niches primarily in the DON and auricular regions. This variation highlights species-specific adaptations in neural development and organization. At the very posterior end of the *T. ocellata* brain, in the *Lobus electricus (LE)*, a little ventricle was still present, and it maintained positivity to the Msi1 signal. We were even able to find a pH3+/PCNA+ cell, a sign that, even if in smaller proportions, adult neurogenesis is still present in the most caudal region of the *LE* (Figure 21 and Figure 22).

### 3.4. Conclusions and Perspectives

This study provides valuable insights into the phenomenon of adult neurogenesis in chondrichthyans, with a particular focus on the Batoidea group, including *Raja asterias* and *Torpedo ocellata*. Our findings suggest that adult neurogenesis in these species is widespread and potentially represents an ancestral trait within the Chondrichthyan lineage. We observed distinct neurogenic niches in various regions of the brain, including the telencephalon, mesencephalon, and cerebellum, with significant variations between species.

In *R. asterias*, neurogenic activity was prominent across the telencephalon, mesencephalon, and cerebellum, with a marked presence of neurogenic markers such as S100B, Musashi1 (Msi1), and Sox2. The high density of S100B+ cells in the parenchyma, coupled with active cell division, suggests that these cells may contribute to maintaining neural plasticity in the adult brain. Additionally, the multiple neurogenic niches in the cerebellum, particularly in the *corpus cerebelli* and auricles, further emphasize the complexity and plasticity of neural development in *R. asterias*.

In contrast, *T. ocellata* exhibited a more restricted neurogenic pattern in the cerebellum, with fewer niches primarily in the dorsal *octavolateralis* nucleus and auricular regions. Despite a smaller number of niches, *T. ocellata* still showed active neurogenesis, as evidenced by the presence of Msi1+, PCNA+, and pH3+ cells. The existence of neurogenic activity in the caudal region of the *Lobus electricus*, as evidenced by the presence of pH3+/PCNA+ cells, suggests that neurogenesis may play a role even in specialized structures such as the electric organ.

These findings highlight both conserved and species-specific features of adult neurogenesis in chondrichthyans and provide a foundation for future studies aimed at better understanding the cellular and molecular mechanisms underlying neurogenic processes in these ancient vertebrates. Further investigations, particularly in the characterization of different progenitor cell populations and the functional significance of these neurogenic niches, will be essential to fully elucidate the role of neurogenesis in brain plasticity and adaptation across species.

Future studies—which are beyond the scope of the present manuscript—could focus on identifying the molecular pathways that regulate neurogenesis in chondrichthyans, including the role of key signaling pathways such as Notch, Wnt, and fibroblast growth factors in controlling neural progenitor cell behavior. Understanding the interactions between these pathways will be crucial for deciphering the mechanisms that guide the maintenance and differentiation of progenitor cells in the adult brain.

Moreover, examining the functional significance of neurogenesis in different brain regions will provide insights into the ecological and behavioral adaptations of Chondrichthyan species. For instance, how neurogenesis contributes to sensory processing in species like *T. ocellata*, with its electric organ, could lead to a better understanding of how neurogenesis is adapted to specialized brain functions.

Additionally, comparative studies across other Chondrichthyan species and different vertebrate groups will help clarify the evolutionary conservation, or rather the diversity of neurogenic processes. By identifying conserved markers and cell types across species, future research could explore how these mechanisms evolved and how they contribute to brain plasticity in response to environmental demands.

In parallel, further research into the spatial organization and lineage of neural precursors in these species could provide new insights into the dynamics of brain regeneration and plasticity. This is particularly relevant in the context of understanding how neurogenic niches respond to injury or other environmental challenges, which could open up avenues for regenerative medicine applications. Ultimately, these studies could provide new perspectives on the potential for harnessing the regenerative capabilities of the adult brain, not only in chondrichthyans but also in other vertebrate groups, including humans.

## 4. Materials and Methods

### 4.1. Tissue Collection and Preparation

Adult specimens of *R. asterias* and *T. ocellata* were provided fresh by local fishermen. Brains were dissected immediately and fixed in 4% PFA (paraformaldehyde) overnight, in accordance with the approval of the Italian Ministry of Health (cod. B290E.N.TU2). After 24 h in 4% PFA, tissues were transferred to 30% sucrose for cryoprotection and allowed to equilibrate for 24–48 h at 4 °C. Following this period, brains were embedded in OCT (Tissue-Tek O.C.T. Compound; Sakura Finetek, Venice, Italy) and snap-frozen in isopentane. Once embedded, we sectioned the brains into 45 µm slices using a Leica cryostat and mounted them onto Superfrost Plus glass slides (Thermo Fisher Scientific, Monza, Italy)

### 4.2. Total RNA Extraction and cDNA Preparation

We used the RNeasy Mini Kit (Qiagen, Milan, Italy) for total RNA extraction, according to the manufacturer’s protocol. We quantified RNA using an FC-3100 (Nanoready, Hangzhou city, Zhejiang, China) spectrophotometer and checked the quality using agarose gel in RNAse-free conditions. From the total RNA, we performed retrotranscription, synthesizing cDNA using the Reverse Transcriptase Core Kit (Eurogentec, Segrate, Italy).

### 4.3. DIG-Labeled Riboprobe Synthesis

Templates for *R. asterias Notch1*, *Notch3*, and *Sox2* were obtained from cDNA using forward, Fw: 5′-ACGCTGTGAAATGGACATCA-3′ and reverse carrying a T7 promoter sequence on its 5′ end, Re: 5′-TAATACGACTCACTATAGGGCGTTCTGACAGGGTTGACTC-3′ for *Notch1*; forward Fw: 5′-GGATCTGGTGAACAAGTACA-3′ and reverse with T7 promoter, Re: 5′-TAATACGACTCACTATAGGGAATCAGGACGTTCTCAC-3′ for *Notch3;* and forward Fw 5′-CAAGATGCACAACTCGGAGA-3′ and reverse with T7 promoter, Re: 5′-TAATACGACTCACTATAGGGTCCAAGTTCTGTGCTTTGCT-3′ for *Sox2*. PCR products were then purified with the Wizard^®^ SV Gel and PCR Clean-Up System (Promega, Milano, Italy) and verified by Sanger sequencing (Eurofins Genomics, Milano, Italy). We used 50 ng of PCR as a template to transcribe DIG-labeled probes using the T7 polymerase (Thermofisher Scientific, Monza, Italy) and digoxygenated RNTP mix (Roche, Darmstadt, Germany) for 2 h at 37 °C. The resulting DIG-labeled riboprobe was precipitated with 1/10 of the volume of 5 M LiCl and 2.5 volumes of cold ethanol at −20 °C overnight, washed with 70% cold ethanol, resuspended in nuclease-free water, and stored at −80 °C.

### 4.4. Free-Floating In Situ Hybridization (ff-ISH)

ff-ISHs were performed according to [58], with some modifications, as explained in the following paragraph. Briefly, sections (100 µm) were rehydrated in PBS (phosphate buffered saline), detached from the glass slice, and recovered in a 2 mL safelock tube (one section each). Sections were directly prehybridized for 30 min at 66 °C and then incubated with a digoxigenin DIG-labeled probe at 66 °C overnight. Immediately before incubation, the probe was denatured at 80 °C for 3 min. Sections were washed twice for 15 min at 66 °C, first with 2× SSC and then with 0.2× SSC. Sections were then treated with TMN solution (Tris-MgCl2-NaCl buffer) 3 times for 5 min and then stained with BM-Purple (Roche, Darmstadt, Germany). The staining was constantly monitored under a stereomicroscope (M80 Leica, Wetzlar, Germany) equipped with an LED-light O-ring and blocked with 1% PBST (1× PBS + 1% Triton X-100). Once the color was fully developed, sections were postfixed in 4% PFA overnight at 4 °C and coverslipped.

### 4.5. Double Immunofluorescence

To perform double immunofluorescence staining, we began by rinsing the slides with 1× PBS and then proceeded with acid antigen unmasking (citrate buffer, pH 6) for three minutes. The tissue was then blocked with a solution containing 5% BSA (bovine serum albumin) and 0.3% Triton-X 100 in 1× PBS for two hours at room temperature. We incubated the slides overnight at 4 °C with a combination of primary antibodies, each at its proper dilution (Table 2). The following day, we rinsed the slides with PBS 1× three times and incubated them with the required combination of secondary antibodies, diluted 1:500, for two hours at room temperature. We then rinsed the sections again three times in 1× PBS 1×, and nuclei were counterstained with Hoechst 33342 (Invitrogen, Waltham, MA, USA), diluted 1:5000 in 1× PBS for one minute. Finally, the slides were mounted with Fluoroshield mounting medium (Sigma, St. Louis, MO, USA). All incubations were performed in a humid chamber.

### 4.6. Immunofluorescence Using Monovalent Fab Fragment Secondary Antibodies

To use two primary antibodies generated from the same host species simultaneously, we utilized monovalent Fab fragments (AffiniPure Fab Fragment, Jackson ImmunoResearch, West Grove, PA, USA) (Table 2). These monovalent fragments block immunoglobulins, allowing the use of a second primary antibody from the same host. This process involved two successive steps: we first applied the first primary antibody and blocked it with Fab fragments; then, we added the second primary antibody and labeled it with a conventional secondary antibody. The process is similar to standard double immunofluorescence but with a few differences. After incubating the tissue overnight at 4 °C with the first primary antibody and removing the unbound leftover the following day, we applied the appropriate Fab fragment (instead of the secondary antibody) at a 1:400 dilution. After a 2 h incubation at room temperature, we rinsed the Fab fragments and incubated the antibody overnight at 4 °C with the second primary antibody. The following day, we rinsed off the antibody and incubated the tissues with the appropriate secondary antibody, following the standard double immunofluorescence protocol.

### 4.7. Whole Brain Immunofluorescence

The raja asterias whole brain was dissected, fixed in 4% PFA, dehydrated with increasing concentrations of ethanol (i.e., 50% and 70%), and stored at 4 °C until processing. Consequently, the brain was dehydrated in 100% ethanol for 1 h in agitation, chilled in ice for 20 min, and left in agitation at 4 °C in 66% DCM (dichloromethane)/33% ethanol overnight for lipid removal. The next day, it was rehydrated gradually at 80%, 60%, 40%, and 20% for 1 h at room temperature. Once hydrated, the brain was washed with 1× PBS and incubated in 2% PBSGT (1× PBS, 0.2% Gelatin, 2% Triton X-100) at 37 °C for two days. At the end of the blocking process, the brain was incubated with the primary antibodies (anti-PCNA (M0879)) at the proper dilution (Table 2) in 2% PBSGT in agitation at 37 °C for five days. The brain was rinsed six times with 1% PBST for one hour at room temperature and then incubated in 1% PBSGT (1× PBS + 0.2% gelatin + 1% Triton X-100) with the conjugated secondary antibodies for three days at 37 °C. The remaining unbounded antibodies were washed away six times with 1% PBST and left in 1% PBST overnight. In order to clarify the brain, the sample was dehydrated with increasing concentrations of ethanol (20%, 40%, 60%, 80%, and 100%) and incubated with 66% DCM (dichloromethane)/33% ethanol overnight. The next day, the brain was washed twice with 100% DCM for 20 min, transferred to a dark glass tube with 100% DBE (dibenzylether), and incubated at room temperature overnight. On the final day, the DBE was exchanged twice with MACS^®^ Imaging Solution (catalog n° 130-128-511, Miltenyi, North Rhine-Westphalia, Germany) and incubated for three hours until imaging at room temperature.

### 4.8. Imaging

Images of immunofluorescence-stained samples were acquired using a Zeiss LSM900 Airyscan confocal microscope, a Zeiss AxioScan microscope equipped with an Apotome slide, and a Nikon Eclipse Ts2R equipped with a DS-Qi2 camera. The anatomical maps were obtained by imaging several tiles along the x-y axis using the Zeiss Axioscan with a 10× objective. The magnification of single areas was realized using 20× or 40× objectives, acquiring multiple z-plans of each area distanced by the recommended distance and collapsing them together into a Maximum Projection single image using the Zen suite. Images acquired by Nikon were acquired using only a single Z plane. ff-ISH whole-panoramic-view images were acquired with a Nikon Eclipse600 microscope equipped with a DS-Fi3 color camera (Nikon, Tokyo, Japan) supplied with a double-LED lightO-ring. All images were adjusted for contrast and brightness using either the Zen Blue 3. 11 suite or ImageJ 1.54p. Panels were realized in Adobe Photoshop.

Whole-brain immunofluorescence of R. asterias was acquired using the UltraMicroscope Blaze™ light sheet microscope (Miltenyi, North Rhine-Westphalia, Germany) with a 1× objective.

## Figures and Tables

**Table 1:** Summary table of the staining observed in *R. asterias* and *T. ocellata* brains. + indicates the presence of the staining in a good portion of cells in the area; ++ indicates the presence of staining in a wider area, supposedly involving a higher amount of cells (not quantified); - indicates a low presence of stained cells; X indicates the absence of staining in the area.

Marker	Telencephalon		Mesencephalon		Cerebellum		LE	
	*R. asterias*	*T. ocellata*	*R. asterias*	*T. ocellata*	*R. asterias*	*T. ocellata*	*R. asterias*	*T. ocellata*
S100B	++	+	++	-	+	-	X	-
PCNA	+	+	+	+	++	+	X	+
Msi1	+	-	++	+	+	+	X	+
pH3	+	+	+	+	+	+	X	+

**Table 2:** Table of the antibodies used in this work.

Primary Antibody	Producer	Catalog Number	Type	Working Dilution
Msi1	Cell Signaling	D46A8	Monoclonal rabbit	1:100
PCNA	Sigma	p8825	Monoclonal mouse	1:800
PCNA	Dako	M0879	Monoclonal mouse	1:500
pH3	Abcam	ab47297	Polyclonal rabbit	1:500
S100B	GTX129573	Genetex	Polyclonal rabbit	1:500
**Secondary Antibody**	**Producer**	**Catalog Number**	**Type**	**Working Dilution**
AlexaFluor 488 anti-Rabbit	Invitrogen	A11001	Goat IgG	1:500
AlexaFluor 568 anti-Mouse	Invitrogen	A11004	Goat IgG	1:500
Rhodamine Red™-X (RRX) AffiniPure Fab Fragment	Jackson ImmunoResearch	111297003	Goat/IgG (H+L)	1:400

## Data Availability

The original contributions presented in this study are included in the article/Supplementary Materials. Further inquiries can be directed to the corresponding author.

## Supplementary Materials

The following supporting information can be downloaded at https://www.mdpi.com/article/10.3390/ijms26083563/s1.

## Abbreviations

The following abbreviations are used in this manuscript:

*R. asterias*	*Raja Asterias*
*T. ocellata*	*Torpedo ocellata*
*S. canicula*	*Scyliorhinus canicula*
PCNA	Proliferating Cell Nuclear Antigen
Msi1	Musashi-1
pH3	Phosphorylated Histone H3
SGZ	SubGranular Zone
SVZ	SubVentricular Zone
RGs	Radial Glial cells
MON	Medial Octavolateralis Nuclei
DON	Dorsal Octavolateralis Nuclei
*LE*	*Lobus Electricus*
TAP	Transient Amplifying Progenitors
Tel	Telencephalon
Die	Diencephalon
Mes	Mesencephalon
Crb	Cerebellum
Rho	Rhombencephalon
