# Supplementary material for: Comparative Analysis of Localization and Composition of Adult Neurogenic Niches in the Chondrichthyans Raja asterias and Torpedo ocellata
Source: Int J Mol Sci. 2025 Apr 10;26(8):3563. doi: 10.3390/ijms26083563 (PMC12027359; doi:10.3390/ijms26083563)
Supplement: Supplementary file 1 [file ijms-26-03563-s001.zip › Supplementary Figs.pdf]

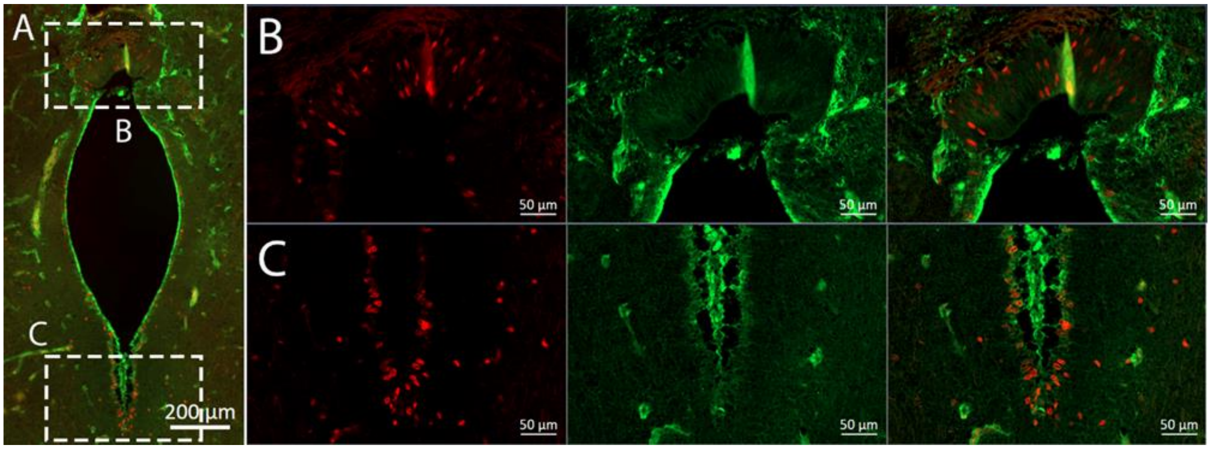

Supplementary Figure S1. Sections of *Raja asterias* Mesencephalon labelled for S100B and PCNA. (A) Overview on the ventricle; (B) Dorsal area of mesencephalic ventricle; (C) Ventral area of mesencephalic ventricle.

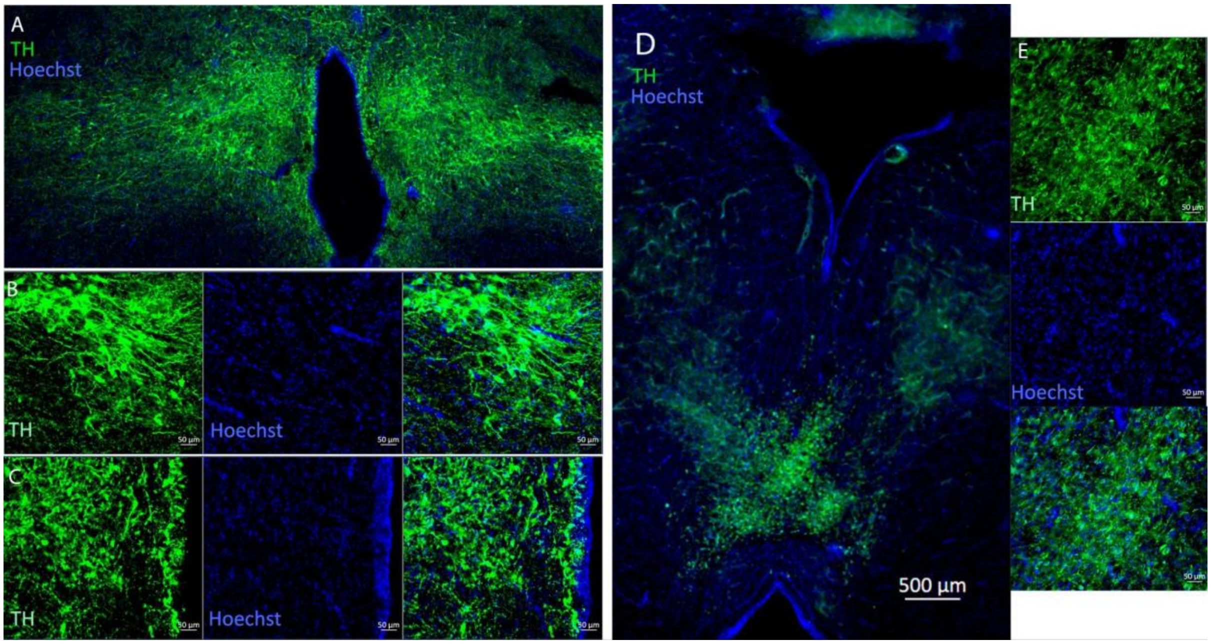

Supplementary Figure S2. Sections of *Raja asterias* Mesencephalon labelled for TH. (A) Overview on the ventricle; (B) Magnification of TH+ cells localized in the mesencephalic parenchima (C) Magnification of TH+ cells localized in proximity of the mesencephalic niche; (D) Overview of the ventral area of the mesencephalic niches. In green are visible TH+ cells localized in the diencephalon; (E) Magnification of TH+ positive cells in the diencephalic area.

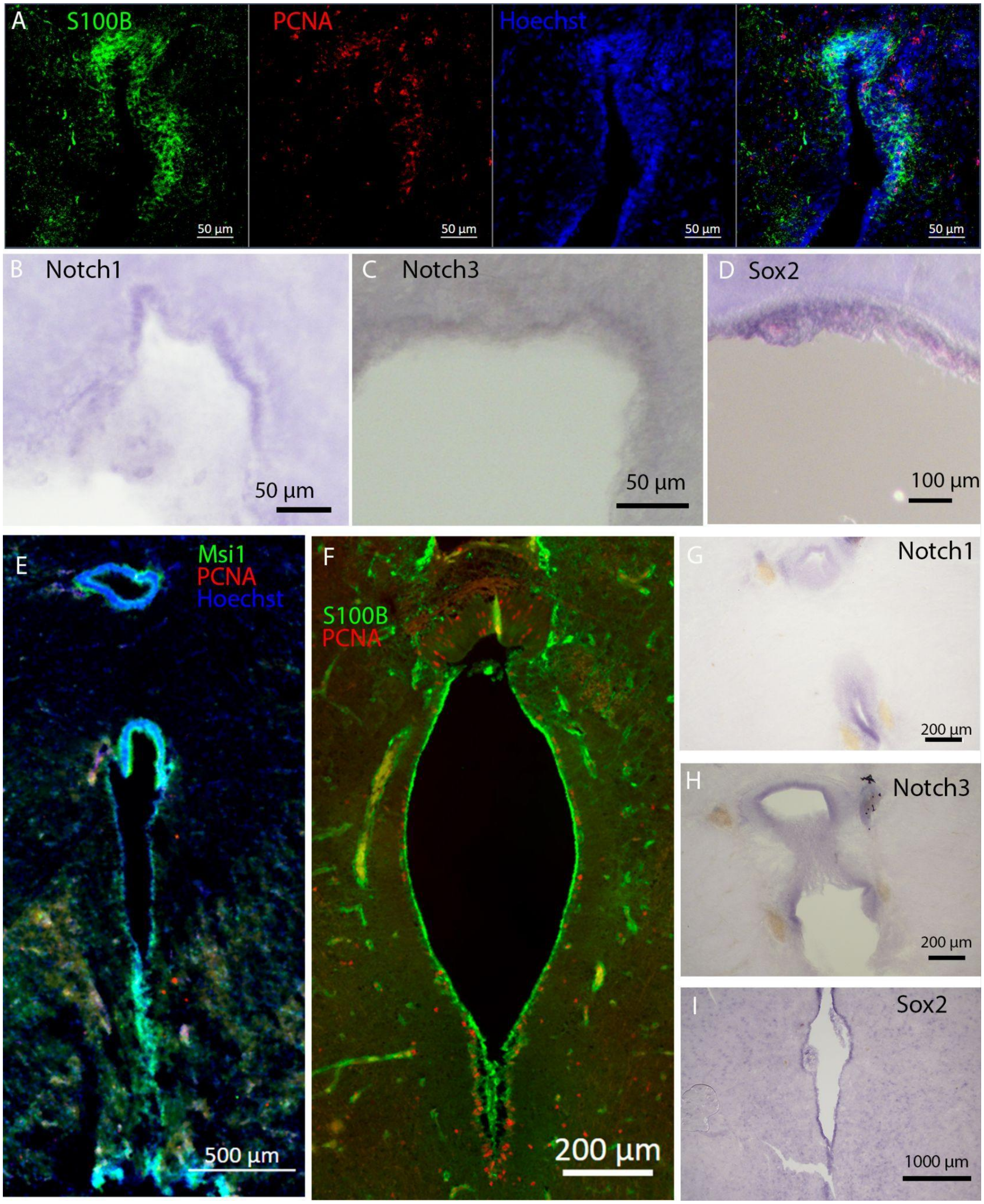

Supplementary Figure S3. Specificity of the *in situ* hybridization stainings. (A) Immunofluorescence for S100B (green) and PCNA (red) realized on R. *asterias* Telencephalon. The section shown is adjacent to the sections utilized for *in situ* hybridization for *Notch1*, *Notch3* and *Sox2*. (B) *In situ* hybridization for *Notch1* in the Telencephalon. (C) *In situ* hybridization for *Notch3* in the Telencephalon. (D) *In situ* hybridization for *Sox2* in the Telencephalon. (E) Immunofluorescence for Msi1 (green) and PCNA (red) in the Mesencephalon. (F) Immunofluorescence for S100B (green) and PCNA (red) in the Mesencephalon. (G) *In situ* hybridization for *Notch1* in the Mesencephalon. (H) *In situ* hybridization for *Notch3* in the Mesencephalon. (I) *In situ* hybridization for *Sox2* in the Mesencephalon.

Supplementary Video S1. Whole cerebellum and optic tectum immunofluorescence of *R. asterias* stained for PCNA (Red).

Supplementary Video S2. Z-stack recording showing PCNA+ neurogenic niche (red) in *Raja asterias* cerebellum and optic tectum .

Supplementary Video S3. Z-stack recording showing PCNA+ neurogenic niche (red) in *Raja asterias* telencephalon.
