# Supplementary figures and images for: Comparative Analysis of Localization and Composition of Adult Neurogenic Niches in the Chondrichthyans Raja asterias and Torpedo ocellata
Source: Int J Mol Sci. 2025 Apr 10;26(8):3563. doi: 10.3390/ijms26083563 (PMC12027359; doi:10.3390/ijms26083563)

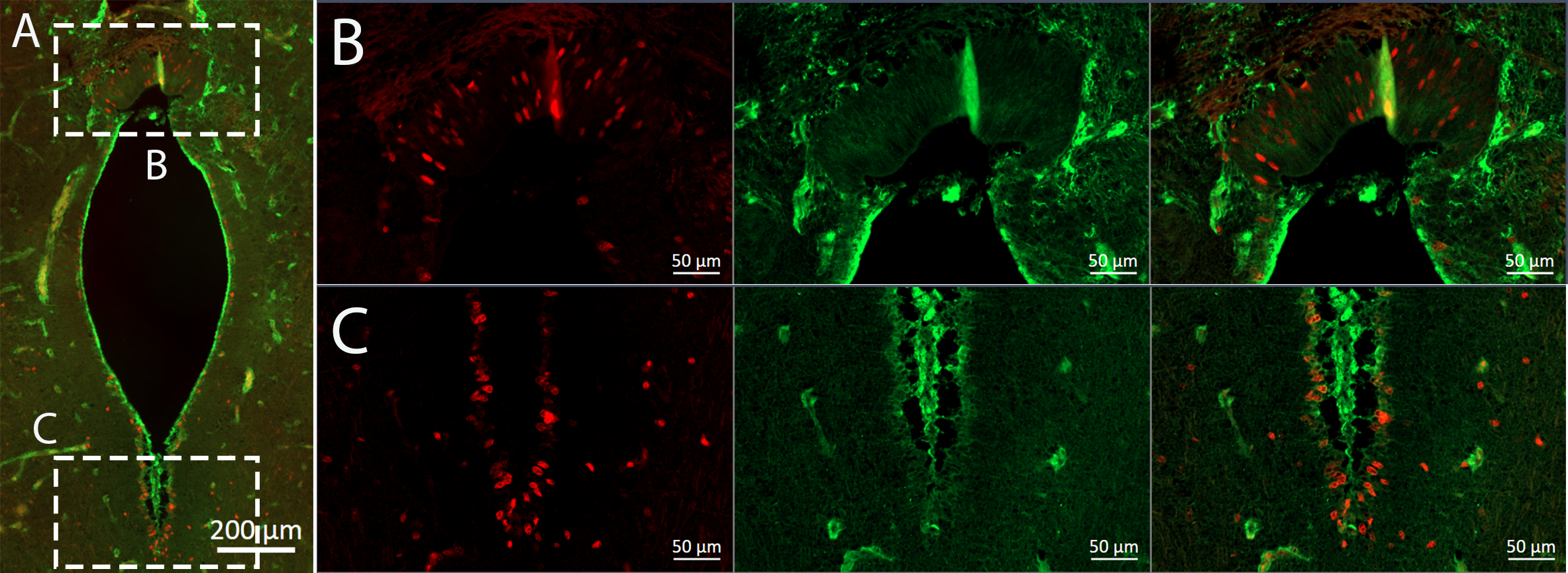

Supplement: Supplementary file 1 [file ijms-26-03563-s001.zip › Supplementary1_MesS100pcna.tif]

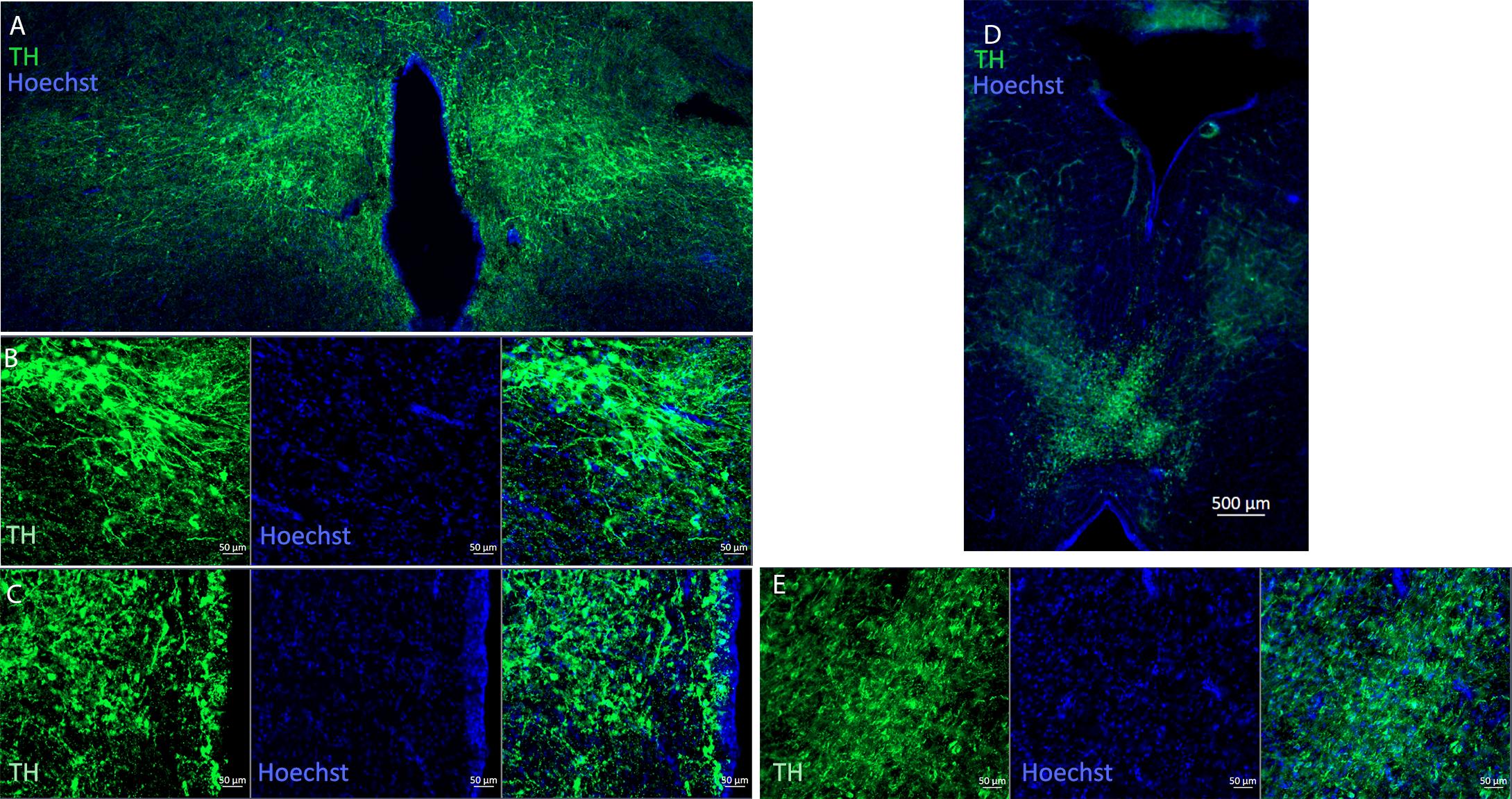

Supplement: Supplementary file 1 [file ijms-26-03563-s001.zip › Supplementary2_THstainingTO SB.tif]

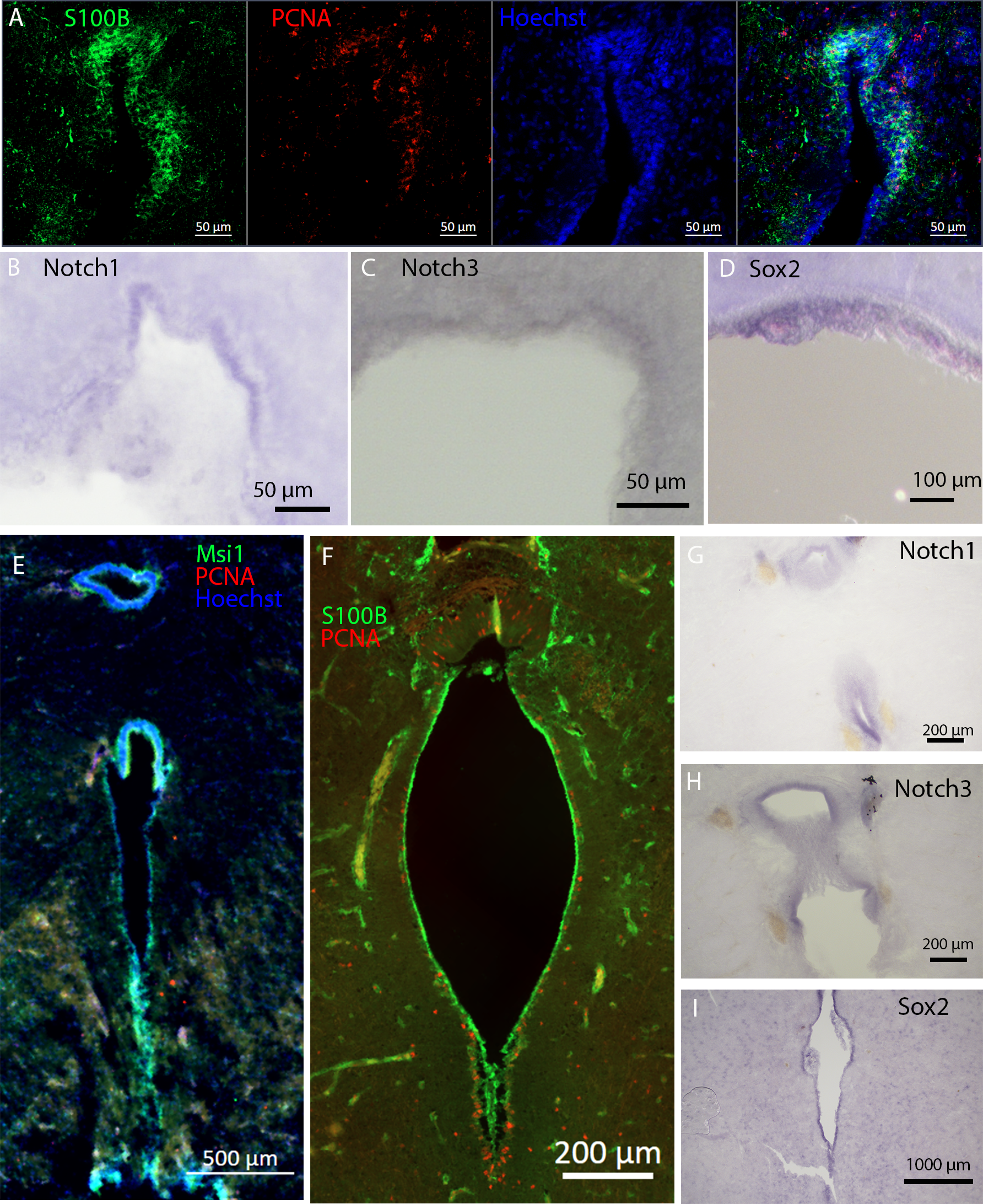

Supplement: Supplementary file 1 [file ijms-26-03563-s001.zip › Supplementary3_TelMesISHImmuno.tif]
